# Transcriptome Analysis Highlights Defense and Signaling Pathways Mediated by Rice *pi21* Gene with Partial Resistance to *Magnaporthe oryzae*

**DOI:** 10.3389/fpls.2016.01834

**Published:** 2016-12-08

**Authors:** Yu Zhang, Jianhua Zhao, Yali Li, Zhengjie Yuan, Haiyan He, Haihe Yang, Haiyan Qu, Chenyan Ma, Shaohong Qu

**Affiliations:** State Key Laboratory Breeding Base for Zhejiang Sustainable Pest and Disease Control, Institute of Virology and Biotechnology, Zhejiang Academy of Agricultural SciencesHangzhou, China

**Keywords:** rice blast disease, durable resistance, *pi21*, partial resistance, RNA-seq

## Abstract

Rice blast disease is one of the most destructive rice diseases worldwide. The *pi21* gene confers partial and durable resistance to *Magnaporthe oryzae*. However, little is known regarding the molecular mechanisms of resistance mediated by the loss-of-function of *Pi21*. In this study, comparative transcriptome profiling of the *Pi21*-RNAi transgenic rice line and Nipponbare with *M. oryzae* infection at different time points (0, 12, 24, 48, and 72 hpi) were investigated using RNA sequencing. The results generated 43,222 unique genes mapped to the rice genome. In total, 1109 differentially expressed genes (DEGs) were identified between the *Pi21*-RNAi line and Nipponbare with *M. oryzae* infection, with 103, 281, 209, 69, and 678 DEGs at 0, 12, 24, 48, and 72 hpi, respectively. Functional analysis showed that most of the DEGs were involved in metabolism, transport, signaling, and defense. Among the genes assigned to plant—pathogen interaction, we identified 43 receptor kinase genes associated with pathogen-associated molecular pattern recognition and calcium ion influx. The expression levels of brassinolide-insensitive 1, flagellin sensitive 2, and elongation factor Tu receptor, ethylene (ET) biosynthesis and signaling genes, were higher in the *Pi21*-RNAi line than Nipponbare. This suggested that there was a more robust PTI response in *Pi21*-RNAi plants and that ET signaling was important to rice blast resistance. We also identified 53 transcription factor genes, including WRKY, NAC, DOF, and ERF families that show differential expression between the two genotypes. This study highlights possible candidate genes that may serve a function in the partial rice blast resistance mediated by the loss-of-function of *Pi21* and increase our understanding of the molecular mechanisms involved in partial resistance against *M. oryzae*.

## Introduction

Rice blast, which is caused by the fungal pathogen *Magnaporthe oryzae*, is the most destructive rice (*Oryza sativa* L.) disease worldwide, leading to 30% of rice yield loss (Skamnioti and Gurr, 2009). Improving disease resistance in rice is crucial for stable food production. Breeding for rice cultivars with durable resistance to rice blast is considered to be one of the most economical, environmentally safe, and effective strategies for disease management (Boyd et al., 2013).

Plants have evolved sophisticated mechanisms to defend against various pathogen invasions, including two main layers of innate immune systems, namely, pathogen-associated molecular pattern (PAMP)-triggered immunity (PTI), and effector-triggered immunity (ETI). PTI is mediated by pattern recognition receptors (PRRs) that recognize highly conserved PAMPs, thereby triggering a relatively weak immune response that restricts colonization by invading organisms. Without race specificity, PTI is predicted to confer durable and broad-spectrum resistance. ETI is mediated by polymorphic resistance proteins that can recognize the highly variable effectors derived from the pathogen, with a markedly fast and strong response usually associated with a hypersensitivity reaction. Owing to the dynamic changes in race (pathotype) and composition of the blast pathogen, the resistance mediated by ETI is generally more specific and predicted to be less durable (Jones and Dangl, 2006).

To date, approximately 100 rice resistance genes conferring resistance to *M. oryzae* have been identified and mapped in the rice genome, and about 25 of them have been cloned (http://www.ricedata.cn/gene/gene_pi.htm; Fukuoka et al., 2014). Blast resistance (R) genes that have been cloned to date belong to the race-specific nucleotide binding site and leucine-rich repeats gene family (NBS-LRR); one exception is *Pi-d2*, which encodes a receptor-like kinase. In contrast to race-specific resistance genes, resistance conferred by quantitative trait loci (QTLs) is characterized by a partial resistance phenotype, usually without race specificity interaction. QTL-mediated resistance is generally more durable than resistance conferred by single race-specific genes, possibly because of decreased selection pressure against the pathogen (Fukuoka et al., 2009). Extensive genetic mapping has highlighted several chromosomal regions harboring beneficial QTLs. Among these, only four genes *pi21, Pb1, Pi63*, and *Pi35* have been cloned (Fukuoka et al., 2009, 2014; Hayashi et al., 2010; Xu et al., 2014). The panicle blast resistance gene *Pb1* encodes an atypical CC-NBS-LRR protein different from previously reported R-proteins, particularly in the NBS domain, in which the P-loop was apparently absent and some other motifs had degenerated (Hayashi et al., 2010). *Pi35* is allelic to *Pish*, which mediates race-specific resistance to blast and encodes a NBS-LRR protein (Fukuoka et al., 2014). A combination of multiple functional polymorphisms in *Pi35* confers durable resistance to blast. *Pi63* gene, which is allelic to *qBR4-2b*, encodes a typical CC-NBS-LRR protein whose transcript expression level is associated with the level of blast resistance (Xu et al., 2014).

*Pi21* encodes a cytoplasmic proline-rich protein that consists of a putative heavy metal-binding domain and a protein—protein interaction motif. This gene represents a susceptibility factor, and loss-of-function of *Pi21* results in durable and race non-specific rice blast resistance. An indicator of hyphal growth, namely, the rate of hyphae penetration from penetrated cells into adjacent cells, is significantly lower in *pi21* plants compared with *Pi21* plants, suggesting that the susceptible *Pi21* allele negatively regulates resistance. Response in resistant *pi21* plants after pathogen attack is not as fast or as strong as the R gene response. The slow induction of defense or incomplete resistance may contribute to the durability of a plant's resistance (Fukuoka et al., 2009). Despite the identification and cloning of *pi21*, the mechanism of the loss-of-function of *Pi21*-mediated resistance, and that of the durable and partial blast resistance in rice is largely unknown.

The transcription regulation of host genes is the central part of plant defense response. Transcriptome sequencing is an effective approach to study gene regulation and signaling network during plant defense response. RNA-seq was used to dissect the transcription regulation and molecular processes in the rice cultivar GV carrying durable and broad resistance (Bagnaresi et al., 2012). GV exhibits a marked upregulation of genes encoding diterpene phytoalexin biosynthetic enzymes, flavin-containing monooxygenase, class I chitinase, and glycosyl hydrolase 17. Chitin oligosaccharide sensing factors, wall-associated kinases, MAPK cascades, and WRKY transcription factors (TFs) were critically involved in defense perception and signaling process in GV. Wei et al. (2013) analyzed the transcriptional profiles of rice early response to *M. oryzae* mediated by R gene by using microarray and identified OsWRKYs as important regulators of rice blast resistance. Recently, comparative transcriptomic analysis of the durably resistant rice cultivar Digu and the susceptible cultivar Lijiangxintuanheigu was conducted to investigate rice early response to *M. oryzae* (Li et al., 2015). Molecular events, including extracellular recognition and biosynthesis of antioxidants, terpenes, and hormones, are specifically activated in Digu before the full maturation of *M. oryzae* appressorium, and the overexpression of a membrane-associated receptor kinase results in enhanced blast resistance. Despite providing important clues to the mechanism of rice blast resistance, the above mentioned studies are mostly focused on rice-*M. oryzae* interaction mediated by R genes (Wei et al., 2013) or the incompatible rice—*M. oryzae* interaction in a single rice cultivar (Kawahara et al., 2012; Wang et al., 2014). Digu is a useful rice germplasm for blast resistance breeding, but its durable resistance is conferred by more than one resistance gene (Li et al., 2015). Transcriptome and molecular mechanism related to single durable resistance gene are relatively less investigated.

In this study, we generated transcriptome data to compare gene expression profiles of the *Pi21*-RNAi transgenic rice line and Nipponbare (Nip) during the early *M. oryzae* infection stages (0, 12, and 24 h post-inoculation, hpi) until the late stages (48 and 72 hpi). Our objectives were to investigate the genome-wide co-expression of genes in the *Pi21*-RNAi line and to identify potential genes involved in the loss-of-function of *Pi21*-mediated blast resistance. Several major gene families may be involved in rice resistance to *M. oryzae* attack, including receptor-like kinases, WRKY TFs, diterpene phytoalexin biosynthesis genes, and *PR* genes.

## Materials and methods

### Plant materials, fungal materials, and fungal infection

Rice (*Oryza sativa* L. ssp. *japonica* cv. Nipponbare) and *Pi21*-RNAi transgenic plants were used in this study. Rice seeds were surface-sterilized and transferred to one-half-strength Murashige and Skoog medium. After germination, rice seedlings were transplanted into soil and kept in a growth chamber at 26/24°C under a 14 h light/10 h dark cycle with 85% humidity. The GUY-11 isolate (Montpellier, France) and TMC-1 isolate (strain E2007046A-2, Hubei, China) of *M. oryzae* were used for inoculation. GUY-11 is compatible with Nipponbare and generates partial susceptible symptoms (Zhou et al., 2006). TMC-1 is compatible with Nipponbare.

Two-week-old *Pi21*-RNAi and Nip rice plants were used for inoculation with GUY-11 and TMC-1. Spore concentration was adjusted to 5 × 10^5^ spores/mL with 0.02% Tween-20. The fungal-inoculated rice seedlings were kept in a dark chamber at 85% humidity and 24°C. After 34 hpi, the plants were maintained in the growth chamber at 26/24°C in a 14 h light/10 h dark cycle with 85% humidity. Leaves were harvested at 0 (immediately after inoculation), 12, 24, 48, and 72 hpi for experimentation. To ensure that inoculation was successfully performed, reverse transcriptional-polymerase chain reaction (RT-PCR) was performed on rice leaf samples collected for the expression of pathogen-related (PR) genes after inoculation with blast, and the remaining seedlings were kept for another 5 days post-inoculation for disease evaluation. The harvested leaves were immediately frozen in liquid nitrogen and kept at −80°C until RNA extraction.

### Generation of *Pi21*-RNAi transgenic plants

The method of RNA-interference-mediated knockdown of *Pi21* was performed. A 275-bp region of the 3′- UTR, starting 17 bp downstream from the stop codon, was used to make a *Pi21* silencing vector as described previously (Fukuoka et al., 2009). The *Pi21* silencing vector construct was transformed via *Agrobacterium* into rice Nipponbare embryogenic callus, and 93 independent transgenic plants were generated. We used T_1_ plants derived from T_0_ transgenic lines with decreased *Pi21* expression confirmed by quantitative RT-PCR (qRT-PCR) analysis and more resistance to blast fungus, as confirmed by inoculation tests.

### Construction of illumina library and sequencing

For Illumina sequencing, total RNAs were isolated from #241 *Pi21*-RNAi and Nip leaves at 0, 12, 24, 48, and 72 hpi with two *M. oryzae* strains by using TRIzol Reagent (Invitrogen, Burlington, ON, Canada) according to the manufacturer's instructions and treated with gDNA eraser (Takara, Japan) to remove any genomic DNA. After the yield and purity of RNA were accessed, the RNA sequencing libraries were constructed and sequenced by using Illumina HiSeq™ 2000 with a 50 bp single-end reads protocol.

### Normalized expression levels of genes from RNA-Seq and gene annotation

For gene expression analysis, ERANGE software (version 4.0) (http://woldlab.caltech.edu/gitweb/) was used to calculate the normalized gene locus expression level by quantifying the number of reads that were mapped to the rice (*Oryza sativa* L. ssp. *japonica* cv. Nipponbare) genome sequences. The expression level of a gene from RNA-Seq was normalized by the reads per kb per million mapped reads (RPKM) method (Mortazavi et al., 2008). Gene annotation, including gene ontology (GO) and Kyoto Encyclopedia of Genes and Genomes (KEGG) annotations, was referred by Zhang et al. (2014). The plant TFDB database was used for analyses of rice TFs, and the rice kinase database was used for the analysis of rice kinase.

### Screening of DEGs

The *R*-package DEGseq was applied to identify DEGs with the random sampling model based on the read count for each gene at different developmental stages (Wang et al., 2010). The false discovery rate (FDR) was used to determine the threshold of *P*-value. A combination of FDR ≤ 0.001 and the absolute value of log_2_ Ratio ≥ 1 were used as the threshold to judge the significance of gene expression difference. For grouping DEGs with similar expression patterns, a hierarchical clustering was generated using the expression values from each library. Analysis was conducted using Cluster 3.0 software with Pearson correlation as the distance measure. The cluster tree contained distinct clusters, which included genes with a unique expression profile by visual inspection. For pathway analysis, we mapped all DEG with more than twofold differential representation to terms in the KEGG database, and then looked for significantly enriched pathway terms compared to the genome background. For a graphical overview of pathways of metabolism and regulation, we used the MapMan tool (http://MapMan.gabipd.org).

### Quantitative real-time PCR (qRT-PCR) analysis

The leaf total RNAs were isolated by TRIzol reagent, and the first-strand synthesis of the cDNAs was carried out by PrimeScript™ RT reagent kit with gDNA eraser (Takara, Japan) according to the manufacturer's instructions. Primers were designed using Primer 5.0 (Premier Biosoft International, CA), and the primers used in the qRT-PCR analysis are listed in Supplementary Table S1. The reference gene elongation factor 1α (EF-1α) was selected for use based on a previous research (Caldana et al., 2007). qRT-PCR was carried out in 384-well plates with an QuantStudio™ 6 Flex Real-Time PCR System (Applied Biosystems, USA) using an SYBR Green Master ROX (Roche, Japan) and following the manufacturer's instructions. All tested samples were technically duplicated and all experiments were performed with three biological replicates. The thermal cycle used was as follows: 95°C for 10 min; 40 cycles of 95°C for 15 s, 58°C for 10 s, and 72°C for 15 s. After the reactions, a melting curve analysis was conducted to evaluate the primer specificity. The relative expression levels of the selected genes, normalized to the reference gene, were calculated using the 2^−ΔΔCT^ method (Livak and Schmittgen, 2001) and a statistical analysis of the expression level of selected genes were performed using Paired *t*-test.

## Results

### Generation of *Pi21*-RNAi transgenic rice line and *M. oryzae* inoculation

**Figure 1:**
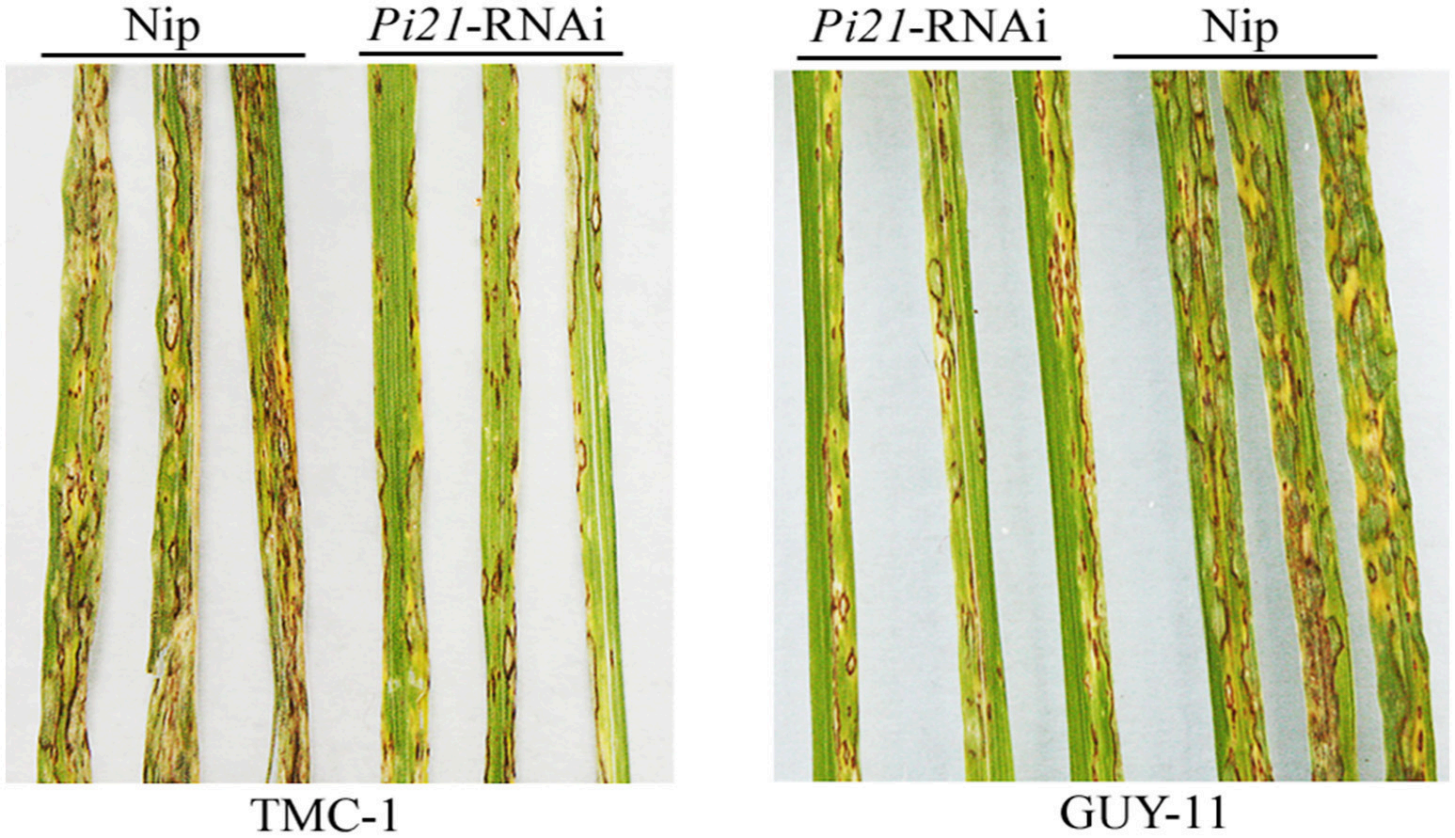
**Disease symptoms of transgenic ***Pi21***-RNAi line and Nipponbare (Nip) leaves at 5 days post-inoculation by isolations of TMC-1 and GUY-11, respectively**. The inoculation experiment was repeated thrice (*n* = 10) with similar results.

A 275-bp region of the 3′ UTR of *Pi21* was used as RNA interference target sequence as described previously (Fukuoka et al., 2009) to make a *Pi21*-RNAi vector. Ninety-three independent transgenic *T*_0_ lines were generated by transforming the *Pi21*-RNAi construct into Nip. Twelve *T*_1_ transgenic lines with a 3:1 segregation ratio were screened using a GFP reporter gene in the *Pi21*-RNAi T-DNA. GFP-positive plants from each *T*_1_ line were subjected to qRT-PCR analysis. The *Pi21* transcript levels in the GFP-positive *T*_1_ plants significantly decreased compared with the untransformed Nip plants (Supplementary Figure S1). T-DNA flanking sequences in eight of the above-mentioned rice lines were isolated using TAIL-PCR. PCR using primers specific to the T-DNA right or left border, and flanking rice sequences surrounding different T-DNA integration site of each transgenic rice line were performed to confirm the junction between the T-DNA and the flanking rice sequences. For the #241 *Pi21*-RNAi line, the junctions between both T-DNA right and left borders and their flanking rice sequences were confirmed, thereby suggesting that the #241 line carried a relatively simple T-DNA insertion than other transgenetic rice lines (Supplementary Figure S2). After inoculation with *M. oryzae* isolates GUY-11 and TMC-1, the #241 *Pi21*-RNAi showed a slow disease response and was more resistant than Nip (Supplementary Table S2; Figure 1). Compared with AA-*pi21* (a near-isogenic line carrying *pi21* in the genetic background of a susceptible cultivar, Fukuoka et al., 2009), #241 showed a similar resistant extent to rice blast and then #241 *Pi21*-RNAi line can be used for transcriptome sequencing. Prior to RNA-seq, RT-PCR analysis of *OsPR1b* (LOC_Os01g28450), *PAL4* (LOC_Os02g41680), and a putative pathogen-related gene (LOC_Os01g53100) were validated in the #241 *Pi21*-RNAi transgenic rice line and Nip infected with GUY-11 and TMC-1 at five time points (Supplementary Figure S3). The induced expression pattern of the *PR* genes at 12 hpi indicates that the rice immune response has been successfully activated. Then, the same batch rice samples as Figure 1 (GUY-11 and TMC-1-infected *Pi21*-RNAi transgenic rice and Nip leaves) were used for transcriptome sequencing.

### Illumina sequencing and data analysis

To study the molecular mechanisms involved in the partial resistance of the loss-of-function of *Pi21* to *M. oryzae*, 20 cDNA libraries from the *Pi21*-RNAi line and Nip leaves at 0, 12, 24, 48, and 72 hpi with two *M. oryzae* strains, respectively, were constructed using total RNA, and a large amount of data was generated with the Illumina HiSeq 2000 platform. A total of 217 million clean reads were obtained from the 20 infected rice libraries. Each library represented by more than 10 million reads, reaching the saturation level of gene identification for the quantitative analysis of gene expression. All clean reads were mapped to the *Oryzae sativa* L. cv. Nip reference genome. For each sample, approximately 84% of the clean reads were successfully aligned to the reference sequences of rice genome (maximum of two mismatches). Among the total clean reads, approximately 80% matched the unique genomic locations and the uniquely matched reads were used for gene expression analysis of each library. An overview of the mapped statistics is provided in Supplementary Table S3. The RNA-seq data were reliable and suitable for further transcriptome analysis. The RNA sequencing data is deposited at SRA website, accession number SRA492222.

**Figure 2:**
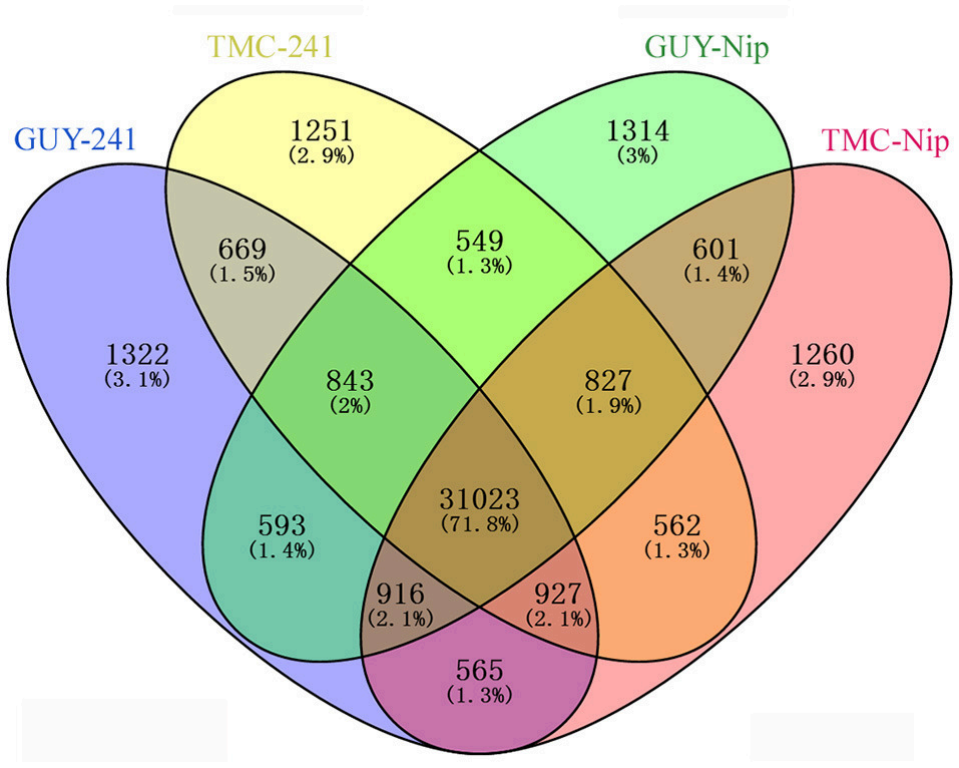
**Venn diagram showing the genes expressed in ***Pi21***-RNAi transgenic line and Nip infected with ***M. oryzae*****. A total of 36,858, 36,651, 36,666, and 36,681 genes were identified from the *Pi21*-RNAi line infected with GUY-11, the *Pi21*-RNAi line infected with TMC-1, Nip infected with GUY-11, and Nip infected with TMC-1, respectively.

Our RNA-seq data generated 43,222 genes that account for approximately 72% (43,222/59,986) of the annotated rice genes in the Nip genome detected at five inoculation time points. In total, 36,858, 36,651, 36,666, and 36,681 genes were identified from the *Pi21*-RNAi line infected with GUY-11, the *Pi21*-RNAi line infected with TMC-1, Nip infected with GUY-11, and Nip infected with TMC-1, respectively. As shown in the Venn diagram, most of the detected genes (71.8%) were observed in both *Pi21*-RNAi and Nip lines; a total of 669 genes were specifically expressed in the *Pi21*-RNAi plants and 601 genes specifically expressed in Nip at one to five time points post-inoculation (Figure 2). Genes that were specifically regulated in the *Pi21*-RNAi line may perform important functions in durable rice blast resistance. Gene expression patterns at five inoculation time points are summarized in Supplementary Table S4.

### Identification of blast infection-related DEGs between the *Pi21*-RNAi line and nip

**Figure 3:**
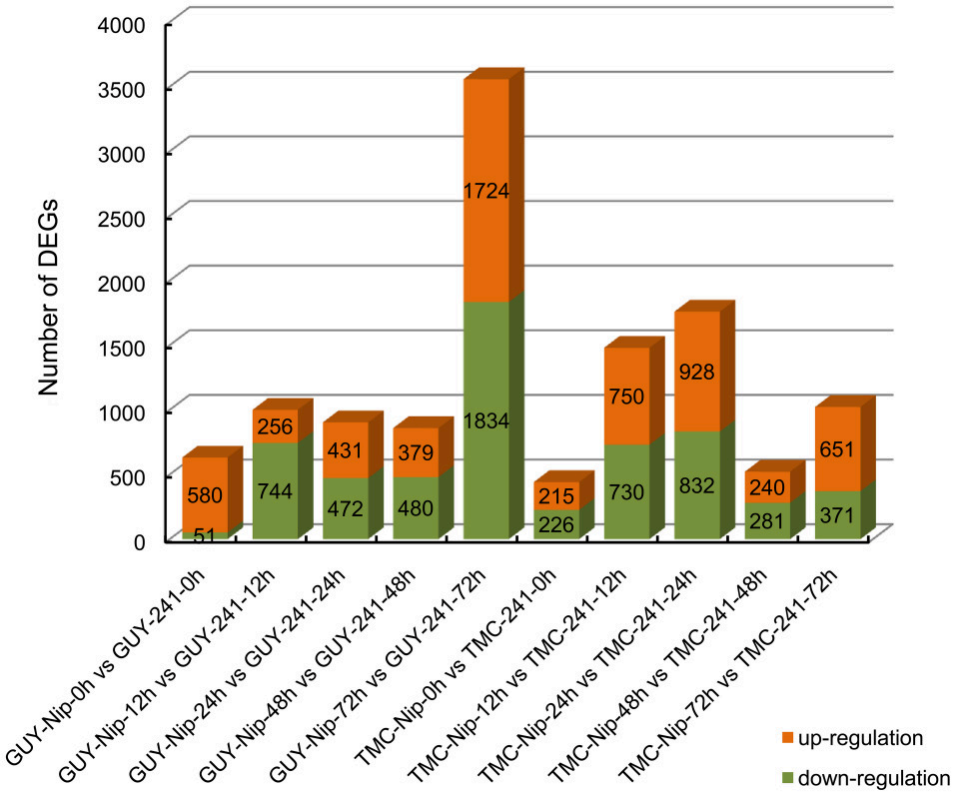
**Differentially expressed genes detected between the ***Pi21***-RNAi line and Nip at five time points**. The numbers of up-regulated and down-regulated genes in the *Pi21*-RNAi line compared to Nip are revealed.

To uncover the genes that may be involved in rice blast infection, DEGs were identified in Nip and the *Pi21*-RNAi line at 0, 12, 24, 48, and 72 hpi with GUY-11 and TMC-1. In our study, DEG was defined as the fold change of the normalized (RPKM) expression values and was at least two-fold. The false discovery rate was ≤ 0.001. Then, a total of 5151 DEGs were identified between Nip and *Pi21*-RNAi line infected with GUY-11 and 3984 DEGs between two genotypes infected with TMC-1, thereby resulting in a union of 6865 DEGs infected with GUY-11 or TMC-1 (Supplementary Table S5). Figure 3 showed that differentially expressed genes were detected between the *Pi21*-RNAi line and Nip at five time points. Compared with Nip, the numbers of up-regulated and down-regulated genes in the *Pi21*-RNAi line were revealed.

**Figure 4:**
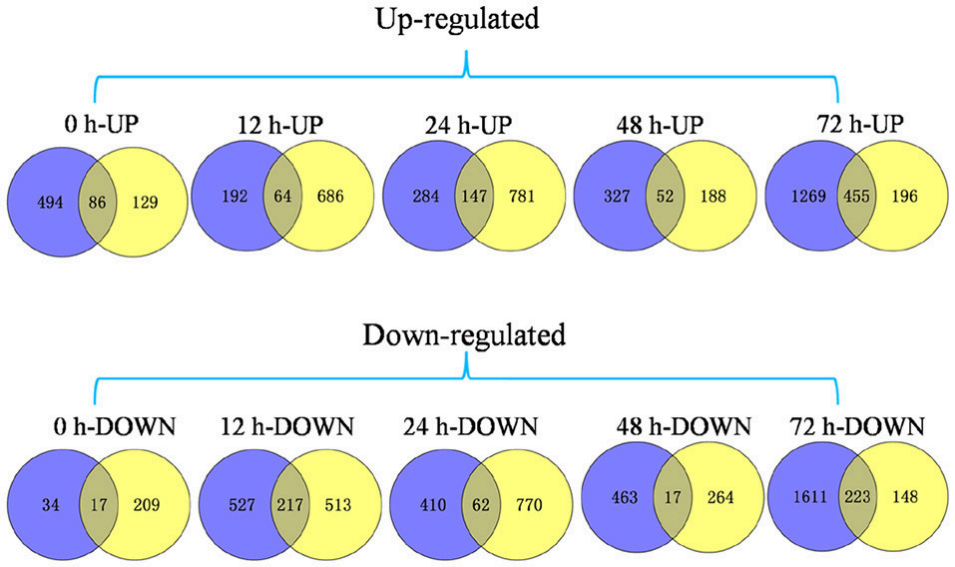
**Venn diagram of DEGs in the ***Pi21***-RNAi line compared with Nip within 72 hpi**. A set of 1109 genes exhibited significant differential expression between two genotypes infected with both isolates. We identified 103 (86 up-regulated and 17 down-regulated) DEGs, 281 (64 up-regulated and 281 down-regulated) DEGs, 209 (147 up-regulated and 62 down-regulated) DEGs, 69 (52 up-regulated and 17 down-regulated) DEGs, and 678 (455 up-regulated and 223 down-regulated) DEGs in the *Pi21*-RNAi line at 0, 12, 24, 48, and 72 hpi compared with that in Nip, respectively. *Blue* represents DEGs between two genotypes infected with GUY-11. *Yellow* represents DEGs between two genotypes infected with TMC-1.

We were particularly interested in genes that were differentially expressed between the *Pi21*-RNAi line and Nip inoculation of both GUY-11 and TMC-1; as such, genes that may be related to partial blast resistance conferred by the loss-of-function of *Pi21*. Correlations between genes obtained with the two isolates were analyzed. Pearson's correlation coefficients of GUY-11-and TMC-1-challenged samples ranged from 0.91 to 0.99 with an average of 0.95, indicating that a similar set of genes were expressed in GUY-11-and TMC-1-challenged samples. As a result, a total of 1109 genes were detected with commonly differential expression in the *Pi21*-RNAi line compared with Nip infected with both isolates in at least one time point (Figure 4). In comparison with Nip, we identified 103 (86 up-regulated and 17 down-regulated), 281 (64 up-regulated and 281 down-regulated), 209 (147 up-regulated and 62 down-regulated), 69 (52 up-regulated and 17 down-regulated), and 678 (455 up-regulated and 223 down-regulated) DEGs in the *Pi21*-RNAi line at 0, 12, 24, 48, and 72 hpi, respectively (Figure 4; Supplementary Table S6).

**Figure 5:**
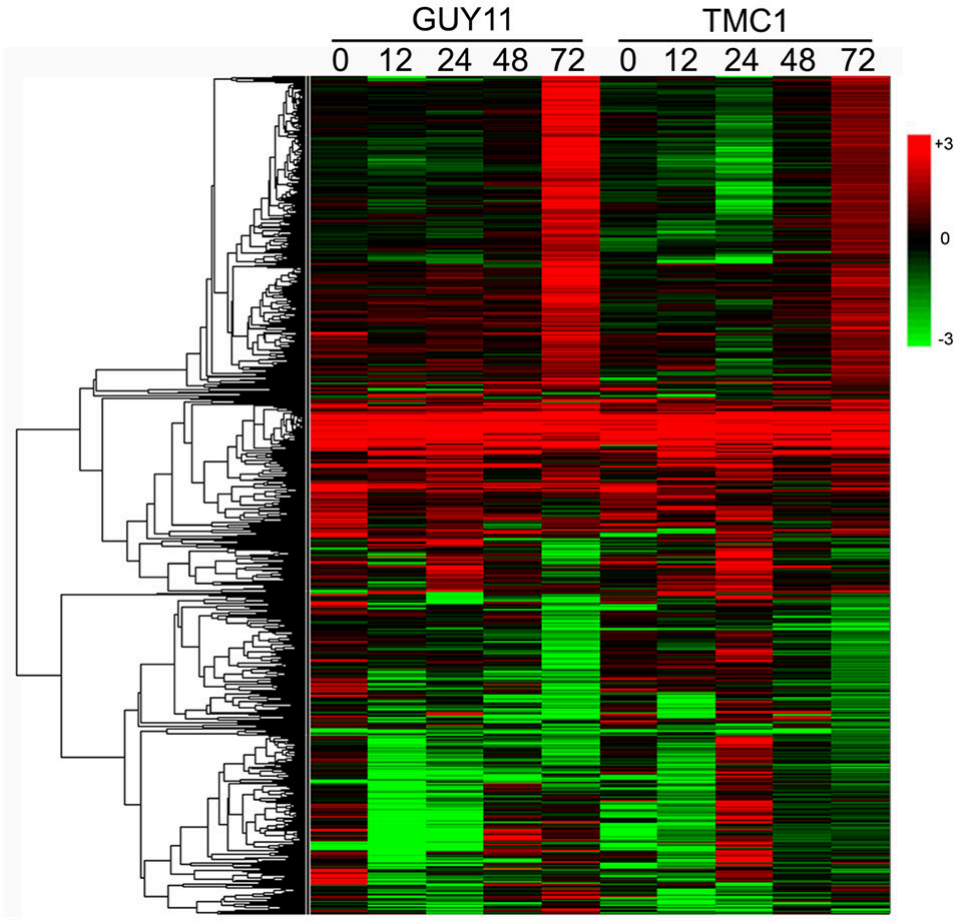
**Hierarchical cluster analysis of 1109 DEGs based on log ratio RPKM data**. The clusters display expression patterns for a subset of 1109 DEGs in the *Pi21*-RNAi line and Nip during GUY-11 and TMC-1 infection. Each column represents the log_2_ fold change in transcript levels in the *Pi21*-RNAi line at the indicated times, relative to the levels of Nip samples. *Red* represents up-regulation expression and *green* represents down-regulation expression. *Each column* represents an experimental condition. *Each row* represents a gene.

Then, we performed hierarchical clustering of the 1109 DEGs. The hierarchical clustering generated a global view of the expression level for DEGs at five time points in the *Pi21*-RNAi line compared with those in Nip. A common set of consecutive regulated DEGs were identified in rice *Pi21*-RNAi line treatments, and these genes may be involved in durable rice defense response to *M. oryzae* (Figure 5).

### GO analysis of DEGs

WEGO (Web Gene Ontology Annotation Plot) analysis of 5151 DEGs between two genotypes challenged by GUY-11 showed that 7, 13, and 15 GO terms were classified in the cellular component, molecular function, and biological process, respectively. WEGO analysis of 3984 DEGs between two genotypes challenged by TMC-1 revealed 22, 32, and 18 functional groups belonging to the cellular component, molecular function, and biological process category, respectively. The overlapping GO terms between the two genotypes infected with GUY-11 and TMC-1 were as follows: “vesicle” (GO:0031982) in the cell component; “catalytic activity” (GO:0003824), “lyase activity” (GO:0016829), and “oxidoreductase activity” (GO:0016491) in the molecular function; and “metabolic process” (GO:0008152), “secondary metabolic process”(GO:0019748), “response to stimulus” (GO:0050896), and “response to stress”(GO:0006950) in the biological process.

Further GO enrichment analysis for 6865 DEGs is shown in Supplementary Table S7. Notably, “oxidoreductase activity,” “terpene biosynthetic and metabolic process,” “diterpenoid metabolic process,” and “isoprenoid metabolic process” were significantly enriched in the *Pi21*-RNAi line relative to Nip at 12, 24, 48, and 72 hpi. Compared with Nip, the GO term involved in “generation of precursor metabolites and energy” was also enriched in the *Pi21*-RNAi line in response to *M. oryzae* infection, indicating that durable resistance may be associated with different metabolism processes and more energy requirement for combating pathogen infection.

**Figure 6:**
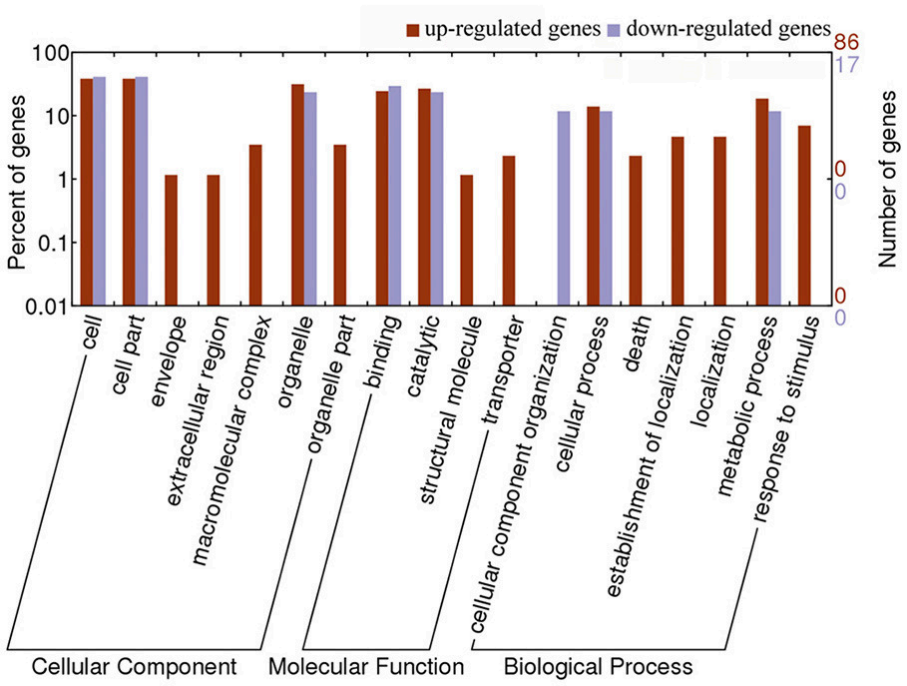
**GO classification analysis of 103 DEGs between the ***Pi21***-RNAi line and Nip at 0 h**. The results are summarized in three main categories: biological process, molecular function, and cellular component by GO analysis.

To investigate the effect of *Pi21*-RNAi on the genes expression in rice, we analyzed the 103 DEGs in the *Pi21*-RNAi compared with Nip at 0 hpi. This set of 103 DEG genes represented an intrinsic difference between the two genotypes. According to WEGO classification, the DEGs could be classified into 18 different functional categories, and most of the DEGs are involved in “cell,” “cell part,” “organelle,” “organelle part,” “metabolic process,” “cellular process,” “response to stimulus,” “binding,” and “catalytic activity” (Figure 6). To identify which biological processes were differentially regulated by knockdown of *Pi21* after *M. oryzae* inoculation, WEGO classification of DEGs at 12, 24, 48, and 72 hpi was analyzed (Supplementary Figure S4). The results show that the GO terms mentioned above were also the prominent GO terms at later four time points. GO enrichment analysis of the DEGs showed that “catalytic activity” was enriched at 12 and 72 hpi. Most likely, certain genes with catalytic activity are involved in rice blast resistance mediated by *pi21*. The results suggest that compared with Nip, “cell,” “cell part,” “organelle,” “organelle part,” “metabolic process,” “cellular process,” “response to stimulus,” “binding,” and “catalytic activity” GO terms exhibit different transcriptional changes in response to *M. oryzae* infection in *Pi21*-RNAi lines.

### Pathway analysis of DEGs

To perform functional classification and pathway assignment of genes that are activated in response to *M. oryzae* invasion, DEGs in each comparison were mapped in the KEGG database. The KEGG enrichment pathways of 6865 DEGs (Figure 3) and 1109 DEGs (Figure 4) between the *Pi21*-RNAi line and Nip are shown in Supplementary Table S8. The “plant—pathogen interaction” and “plant hormone signal transduction” were the top two common enriched pathways, followed by “ribosome” and “nitrogen metabolism” in both genotypes after inoculation with GUY-11 and TMC-1, suggesting that these pathways may perform important functions on basal defense response or plant development. Despite these similarities, careful analysis of the individual genes contributing to the common enriched KEGG pathways revealed substantial diversity between genotypes.

The “metabolic pathways,” “biosynthesis of secondary metabolites,” “stilbenoid, diaryl heptanoid, and gingerol biosynthesis,” and “limonene and pinene degradation” were the enrichment pathways between two genotypes in response to both GUY-11 and TMC-1 infection at 12, 24, 48, and 72 hpi. Besides the four pathways mentioned above, DEGs in “plant—pathogen interaction,” “diterpenoid biosynthesis,” “isoflavonoid biosynthesis,” and “phenylpropanoid biosynthesis” pathways were enriched between two genotypes in response to both GUY-11 and TMC-1 infection at 12, 48, and 72 hpi. Being the *Pi21*-RNAi line-specific or enriched in the *Pi21*-RNAi line relative to Nip, the above-mentioned KEGG pathways are promising candidate genes that underlie *pi21* durable resistance and are listed in Supplementary Table S8. A detailed DEGs examination in the enrichment “plant—pathogen interaction” pathway is shown in Supplementary Table S9. KEGG pathway analysis of DEGs between the *Pi21*-RNAi line and Nip drive the choice of the genes that are subsequently analyzed in details in the following sections.

**Figure 7:**
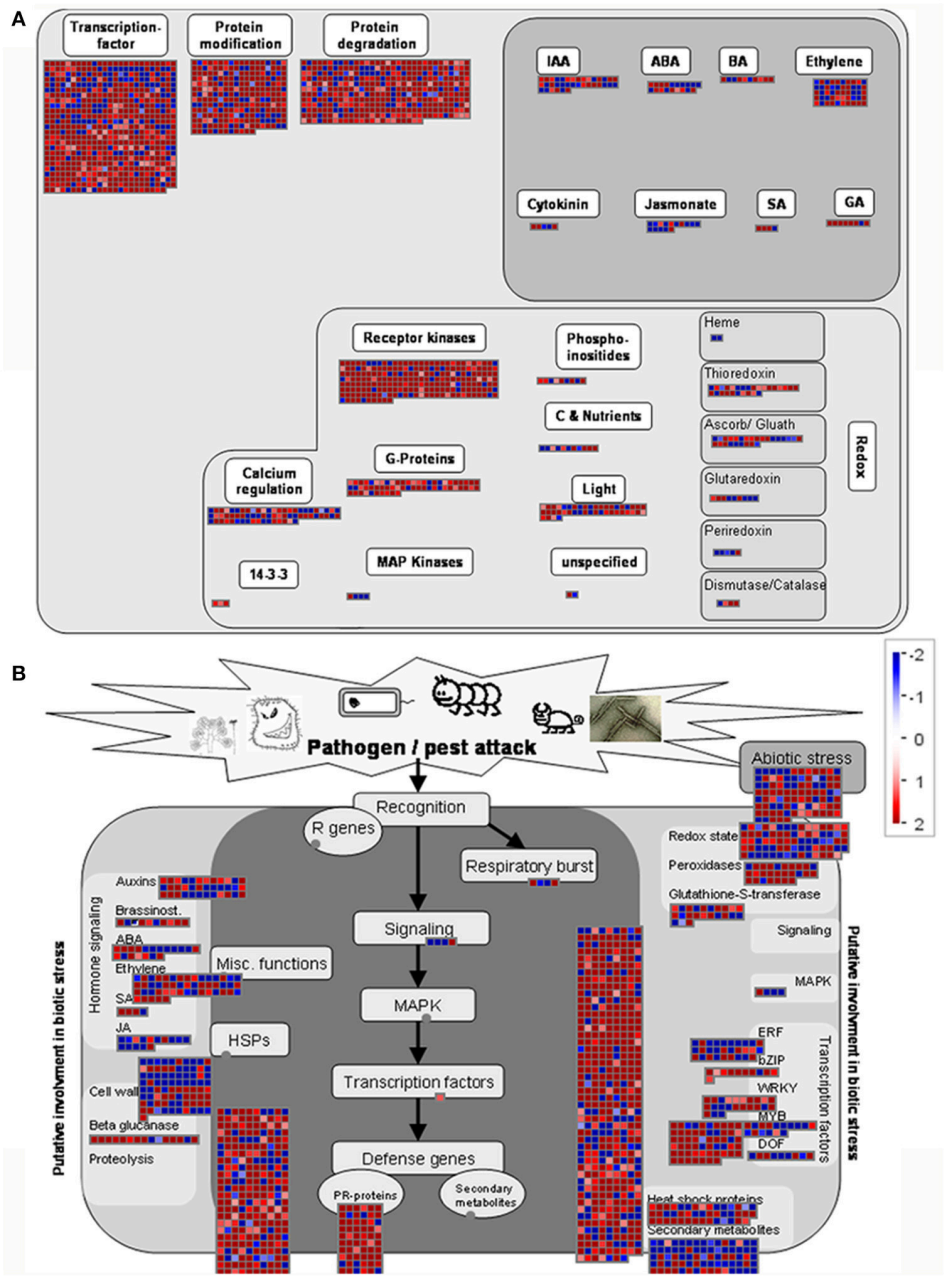
**MapMan overview of regulation (A)** and biotic stress **(B)** showing the transcriptional changes in *Pi21*-RNAi transgenic plants at 12 h post-inoculation. Genes significantly up- (red) or down-regulated (blue) in fungal-inoculated leaf samples relative to 0 h are illustrated. Individual genes are represented by small squares. The scale bar displays log2-transformed fold changes.

**Figure 8:**
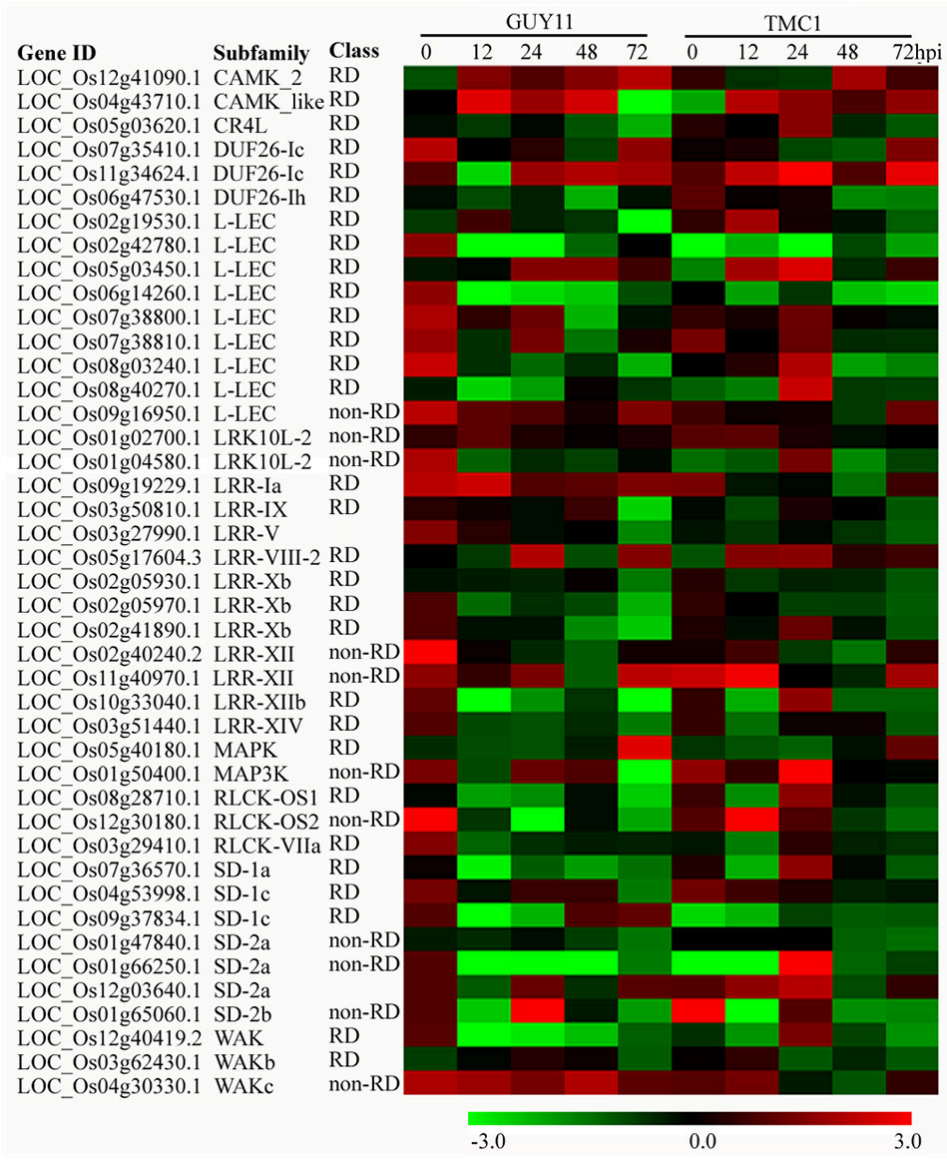
**Heat maps showing expression patterns of receptor kinase (RK) genes that were identified to be differentially regulated in the ***Pi21***-RNAi line compared with Nip**. The IDs of the RK genes were retrieved from the rice kinase database (Dardick et al., 2007). The information on the subfamily and class of the genes was also obtained from the rice kinase database. The RKs encoded by these genes belong to CAMK, domain unknown function 26 (DUF26), lectin-like receptor kinase (L-LEC), LRK10L, leucine-rich receptor (LRR), MAPK, RLCK, cell wall-associated kinase (WAK), and S-domain receptor-like protein kinase (SD) subfamilies. Up- or down-regulated expression of these genes at each time point post-inoculation is indicated. Among these genes, most are up-regulated, specifically in the *Pi21*-RNAi line overall, especially at the point of 24 hpi.

We next used the MapMan package to investigate the pathways involved in rice—*M. oryzae* interaction. The MapMan tool used the input from several experts to curate specific biological processes by using information from the RICE Database. An overview of regulation and biotic stress showing the transcriptional changes in *Pi21*-RNAi transgenic plants at 12 hpi is generated in **Figure 8**. Most of the genes associated with receptor kinases (RKs), calcium regulation, G-proteins, transcription factor, and protein modification/degradation pathways were up-regulated (Figure 7A), indicating that these pathways are important for *M. oryzae* sensing and resistance. Further analyses of biotic stress show that the up-regulated genes were classified into different biological pathways, including signaling, cell wall, redox state, ERF, Myb, WAKY, Zip, PR proteins, proteolysis, and peroxidases. Most of the genes in the SA and ethylene signaling pathways were up-regulated; meanwhile, *LOX2* and *LOX5* genes, which act as JA synthetases, were also up-regulated (Figure 7B). A more detailed list of all DEGs corresponding to MapMan functional categories is provided in Supplementary Table S10. These visual annotations provide a valuable resource for the investigation of pathways involving rice *pi21* gene.

### DEGs related to RKs respond to *M. oryzae* infection

RKs perform important functions as PRRs and regulators of PTI (Kawano and Shimamoto, 2013). To determine whether RKs are involved in the response against *M. oryzae* in the *Pi21*-RNAi line and which RKs were involved, we searched for RKs in the DEGs. A total of 1625 RK genes out of 43,222 genes were identified (Supplementary Table S11).

Forty-three genes encoding protein kinases were differentially expressed in the *Pi21*-RNAi line compared with Nip, and 41 were responsive to *M. oryzae* infection (Figure 8). Among these genes, most were up-regulated in the *Pi21*-RNAi line overall, especially at the points of 0 and 24 hpi. Three RK DEGs, including LOC_Os09g19229.1 (LRR-IA subfamily; RD class), LOC_Os11g40970.1 (LRR-XII subfamily; non-RD class), and LOC_Os04g53998.1 (SD-1c subfamily; RD class), were all up-regulated in the *Pi21*-RNAi line compared with Nip at 0 hpi. This finding suggests that higher expression of the RK genes may perform positive regulatory functions in *pi21* signaling and function. The expression of LOC_Os04g43710.1, which belongs to CAKM-like subfamily, was induced at higher levels in the *Pi21*-RNAi line compared with Nip at 12 and 24 hpi. Two genes encoding LRK10L-2 protein (LOC_Os01g02700.1; non-RD) and WAKc protein (LOC_Os04g30330.1; non-RD) were up-regulated, whereas the other 11 genes were down-regulated in the *pi21* line compared with Nip at 12 hpi. Six RK DEGs, including three L-LEC genes (LOC_Os05g03450.1, LOC_Os07g38800.1, and LOC_Os07g38810.1), one MEKK_ste11_MAP3K gene (LOC_Os01g50400.1), one LRR-VIII-2 gene (LOC_Os05g17604.3), and one SD-2a gene (LOC_Os12g03640.1), all exhibited greater induced of the *Pi21*-RNAi line than compared with Nip at 24hpi. The expression level of LOC_Os08g14950 (LRR-XII) was up-regulated, and the other three genes (LOC_Os06g47530.1, DUF26-1h LOC_Os06g14260.1, L-LEC; *LP2*, LOC_Os02g40240.2, LRR-XII) were down-regulated in the *Pi21*-RNAi line compared with Nip at 48 hpi. Four genes, including *OsSTN8* (LOC_Os05g40180.1), two DUF26-Ic genes (LOC_Os07g35410.1, LOC_Os11g34624.1), and one L-LEC gene (LOC_Os09g16950.1, non-RD), showed more marked inducement in *Pi21*-RNAi plants compared with Nip at 72 hpi. These RKs may be important for signal perception or signal transduction.

### Diterpene phytoalexin genes transcriptionally accumulate in the *Pi21*-RNAi plants respond to *M. oryzae* infection

Fifteen genes involved in the biosynthesis of DPs have been identified in rice. The accumulation of two momilactones and five phytocassanes increased in rice in response to blast fungus infection, accompanied by increased transcription of a minimum of DP biosynthetic genes, as follows: *CPS2, KSL7, KSL10, CPS4, KSL4*, and *KSL8* (Hasegawa et al., 2010). The expression pattern of diterpenoid phytoalexin biologic genes after blast infection in the *Pi21*-RNAi line and Nip is shown in Supplementary Table S12. Among the expression levels of 15 genes, the expression levels of *MAS* (LOC_Os04g10010.1), *KSL7* (LOC_Os02g36140.2), *KSL4* (LOC_Os04g10060.1), *KSL8* (LOC_Os11g28530.2), and *CYP76M8* (LOC_Os02g36070.1) were strongly induced in the two genotypes at 12 hpi, thereby showing 10- to 400-fold increase compared with those at 0 hpi. The expression of most diterpene genes was low at 24 hpi, subsequently increased from 48 hpi, and peaked at 72 hpi. Moreover, *CYP76M7, CYP701A8*, and *CYP99A3* appeared as up-regulated DEGs only in the *Pi21*-RNAi line and as non-DEGs in Nip at 24 and 48 hpi. Rice diterpene phytoalexin accumulation may be critical to the defense responses to blast infection in rice.

### DEGs related to TFs respond to *M. oryzae* infection

**Figure 9:**
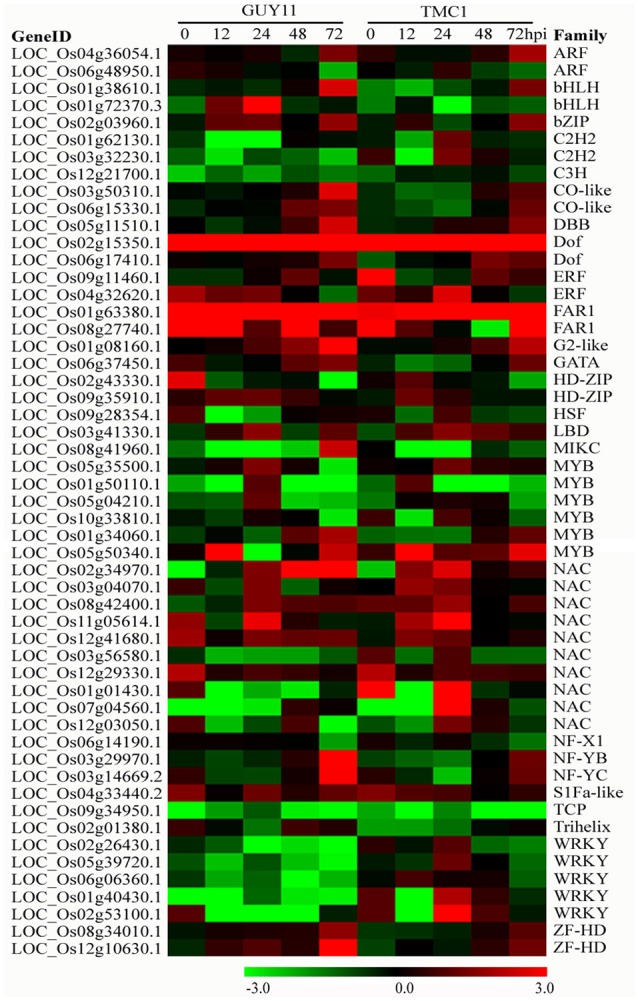
**Heat maps showing expression patterns of transcription factor (TF) genes that were identified to be differentially regulated in the ***Pi21***-RNAi line compared with Nip**. The gene IDs and family names of the transcription factors were obtained from the database of plant TFDB.

TFs are master regulators of gene expression and perform important functions in the transcriptional reprogramming of plant defense-responsive genes during pathogen attack. We detected the expressions of 1840 putative TF genes that could be classified into 55 TF families (Supplementary Table S13). Among 1109 DEGs between the two genotypes infected with both isolates, 53 DEGs were identified as TF genes, which may participate importantly in regulating *pi21* signaling and function (Figure 9). Among the TF DEGs, one FAR1 (LOC_Os08g27740.1), one NAC (LOC_Os12g29330.1), one ERF (*OsRap2.6*, LOC_Os04g32620.1), and one S1Fa-like TF (LOC_Os04g33440.2) were up-regulated, whereas one C3H (LOC_Os12g21700.1) and one bHLH (*OsIRO2*, LOC_Os01g72370.3) were down-regulated in the *Pi21*-RNAi line relative to Nip at 0 hpi. At 12 hpi, one HD-Zip (LOC_Os09g35910.1) was up-regulated, whereas two C2H2, one HSF, three NAC, and two WRKY were down-regulated in the *Pi21*-RNAi line compared with Nip. At 24 hpi, five NAC, one LBD, one Dof, and one Myb were activated to higher expression levels in the *Pi21*-RNAi line relative to Nip. Four TFs (LOC_Os02g15350.1, Dof; LOC_Os09g11460.1, ERF; LOC_Os01g63380.1, FAR1; LOC_Os09g34950.1, TCP) exhibited significantly different expression levels between the *Pi21*-RNAi line and Nip at 48 hpi. At 72 hpi, 27 DEGs were detected, most of which belong to Myb, NAC, WRKY, and Dof TF families. In brief, 30 TFs were up-regulated in the *Pi21*-RNAi line but down-regulated or exhibited no significant changes in the Nip line after *M. oryzae* infection; thus, the TFs may positively regulate rice immunity against the pathogen. Twenty-four TFs whose expression levels were decreased or showed minor changes in the *Pi21*-RNAi line were significantly activated in Nip upon *M. oryzae* infection; thus, the TFs may negatively regulate rice immunity.

### DEGs involved in phytohormone signaling pathways in response to *M. oryzae* infection

Plant hormones, such as salicylic acid (SA), jasmonate (JA), ethylene (ET), gibberellic acids (GAs), and brassinosteroids (BRs), act as signals to trigger and mediate a diverse array of plant immune responses (Liu et al., 2014). In this study, most genes related to SA or ET signaling were induced or suppressed in *Pi21*-RNAi and Nip plants after inoculation with *M. oryzae*. Forty-seven DEGs in the *Pi21*-RNAi line relative to Nip were enriched in the “plant hormone signal transduction” pathway (Supplementary Table S14). Notably, an auxin receptor *GH3.2* (LOC_Os01g55940.1) appeared to be up-regulated in the *Pi21*-RNAi line compared with Nip at 0 and 24 hpi. The *BAK1* genes (LOC_Os05g17604.3, LOC_Os05g03450.1, LOC_Os07g38810.1, and LOC_Os07g38800.1) involving BR signaling, *BRI1* (LOC_Os12g03640.1) related to BR biosynthesis, and *GID1* (LOC_Os05g33940.1) encoding a GA receptor were up-regulated in the *Pi21*-RNAi line compared with Nip at 24 hpi. *OsSAUR12* (LOC_Os02g52990.1), which belongs to the auxin-responsive *SAUR* gene family, and LOC_Os12g04500.1, a member of the two-component response regulator ARR-A family, are expressed significantly higher in the *Pi21*-RNAi line compared with Nip at 48 hpi. In addition, 11 DEGs related to the signaling pathways of GA (LOC_Os09g28640.1), BR (LOC_Os07g35410.1, LOC_Os09g16950.1, and LOC_Os06g16300.1), cytokinine (LOC_Os01g08160.1, LOC_Os02g35180.1), auxin (LOC_Os02g52990.1, LOC_Os04g36054.1, LOC_Os07g02330.1), and abscisic acid (LOC_Os05g39580.1, LOC_Os03g18600.1) were significantly up-regulated in the *Pi21*-RNAi line compared with Nip at 72 hpi.

### Expression of putative rice blast pathogenesis-related (PR) genes

**Figure 10:**
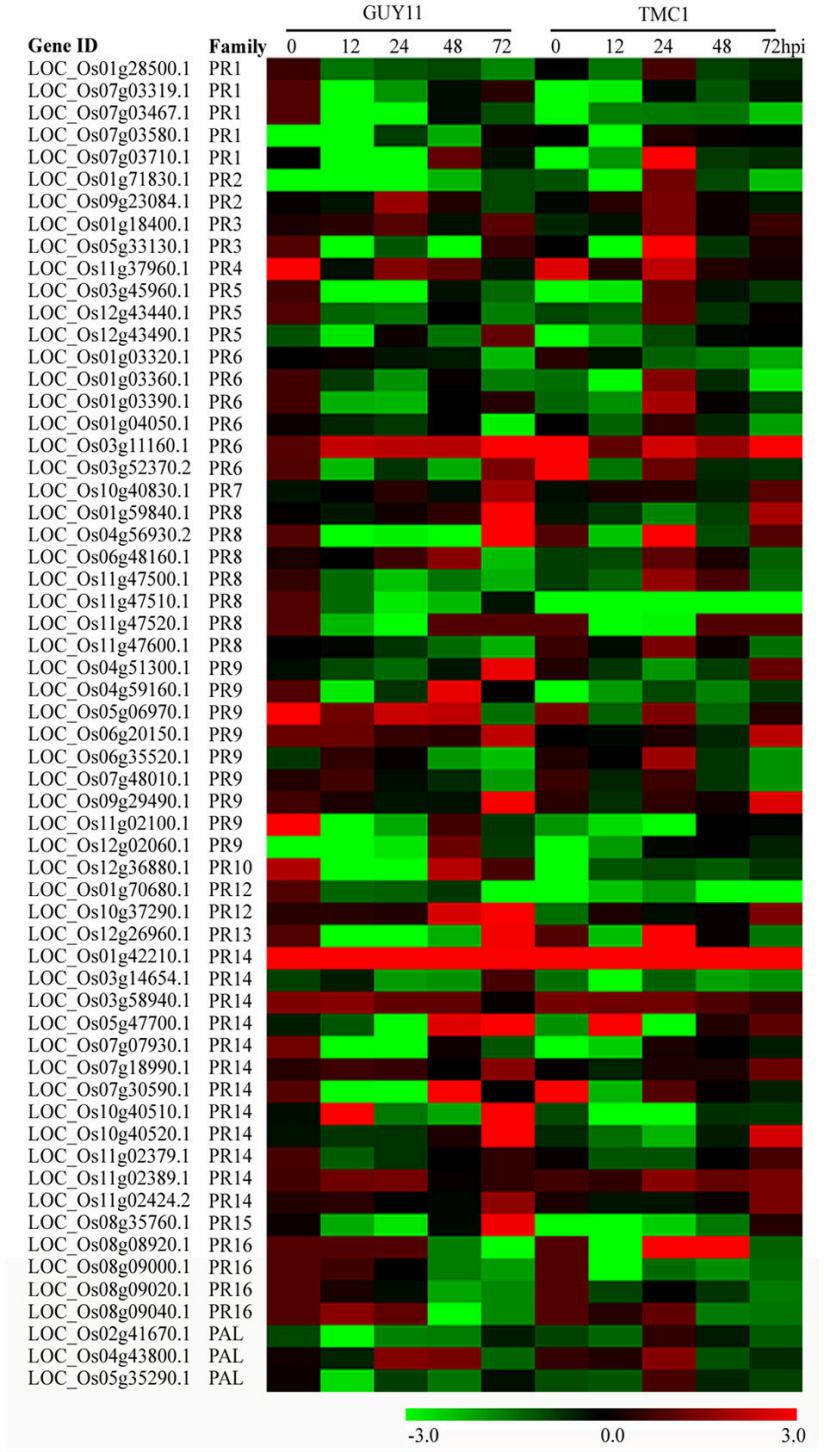
**Heat maps showing expression patterns of pathogenesis-related (PR) and ***PAL*** genes that were identified to be differentially regulated in the *Pi21*-RNAi line compared with Nipponbare**. The gene IDs and family names of the transcription factors were obtained according to Dou et al. (2014).

We investigated the expression of PR genes and their roles in rice defense response to *M. oryzae*. Among 1074 rice PR genes, 274 PR genes were significantly induced by *M. oryzae* in both *Pi21*-RNAi line and Nip. Among the 1109 DEGs derived from the comparison between the two rice genotypes, 62 DEGs (19 up-regulated and 43 down-regulated) were PR genes (Figure 10; Supplementary Table S15). These PR DEGs may be involved in *pi21*-mediated resistance.

The *PR2* gene (LOC_Os09g23084.1, β-1,3-glucanase) exhibited more expression levels in the *Pi21—*RNAi line than that in Nip at 24 hpi. *PR3* (LOC_Os01g18400.1, *OsCHIT10*) and *OsPR4b* (LOC_Os11g37960.1) were higher expressed at 24 hpi in the *Pi21*-RNAi line than that in Nip. *PR8* (LOC_Os01g59840.1) was more induced in the *Pi21*-RNAi line at 72 hpi than that in Nip. The expression of *PR7* (LOC_Os10g40830.1) was more induced in the *Pi21*-RNAi line at 72 hpi than that in Nip. In addition, 4 peroxidase genes (PR9), 1 defensin gene (PR12), and 7 lipid-transfer protein genes (PR14) exhibited significantly different expression patterns between the two genotypes after *M. oryzae* inoculation. These genes were transcriptionally induced at 24 hpi and up-regulated more significantly at 72 hpi than that at 0 hpi in the *Pi21*-RNAi line. By contrast, these genes were moderately suppressed or mildly regulated in Nip. *OsPAL6* (LOC_Os04g43800.1) related to phenylalanine metabolism and phenylpropanoid biosynthesis was up-regulated in *Pi21*-RNAi plants at 24 hpi, whereas *OsPAL6* was repressed in Nip. Taken together, many defense-related genes including *PR* and *PAL* genes showed distinctive expression patterns between the *Pi21*-RNAi line and Nip after *M. oryzae* inoculation. This condition suggests essential roles of these genes in the regulation of quantitative blast resistance of the loss-of-function of *Pi21*.

### Validation of DEGs by qRT-PCR

Fourteen DEGs that might be essential for rice durable blast resistance mediated by knockdown of *Pi21* were selected to validate the DEGs identified by RNA-seq analyses. Moreover, the expression of these genes in response to *M. oryzae* inoculation was investigated by qRT-PCR. The results are shown in Supplementary Figure S5. Of the 14 genes, LOC_Os10g28240.1 encoding a calcium-transporting ATPase, LOC_Os11g44680.1 encoding a calmodulin binding protein, and LOC_Os04g53998.1 encoding a receptor kinase showed a higher expression pattern in the *Pi21*-RNAi line than that in Nip at 0 hpi. LOC_Os10g28350.1 encoding an ET biosynthesis synthase OsARD1 and LOC_Os04g32620.1 (*OsRap2.6*) expressed continuous higher levels in the *Pi21*-RNAi line compared with that in Nip from 0 hpi to 72 hpi. LOC_Os12g12120.1 and LOC_Os11g40970.1 identified as homologs of *FLS2* and *EFR*, respectively, expressed higher levels in the *Pi21*-RNAi line than that in Nip at 0, 12, 24, and 72 hpi. *BAK1* (LOC_Os07g38800.1, LOC_Os12g03640.1, and LOC_Os07g38810.1) exhibited up-regulated expression pattern in the *Pi21*-RNAi line than that in Nip at 24 hpi. LOC_Os11g37960.1 encoding a chitinase and LOC_Os09g23084.1 encoding β-1,3 glucanase also showed a higher expression pattern in the *Pi21*-RNAi line than that in Nip at 24 hpi. LOC_Os05g39720.1 encoding OsWRKY70 showed lower expression levels in the *Pi21*-RNAi line compared with those in Nip after *M. oryzae* inoculation. LOC_Os01g01302.1 encoding shikimate kinases showed higher levels of expression in the *Pi21*-RNAi line than that in Nip at 72 hpi. In general, qRT-PCR data confirm the expression patterns of these important rice blast resistance-related genes revealed by RNA-seq analyses.

## Discussion

In recent years, a wide range of progress has been made in investigating the molecular mechanisms of rice innate immunity, including the related signaling pathways and their role in activating defense responses (Seo et al., 2011; Liu et al., 2014). However, the molecular basis underlying the partial resistance to *M. oryzae* including that conferred by the major QTL *pi21* is still obscure. We performed a comprehensive transcriptomic analysis of the *Pi21*-RNAi line and Nip at different stages of *M. oryzae* infection using RNA-seq to understand the mechanism of the loss-of-function of *Pi21*-mediated partial and durable rice blast resistance.

One rice line generally confers various extent of resistance to different *M. oryzae* strains (Tsunematsu et al., 2000). The gene expression in rice in response to different *M. oryzae* isolates may differ because of the diverse effectors encoded in the isolates and their specific interactions with rice R genes. Defense signaling pathways leading to PTI and ETI are often crosstalk (Kou and Wang, 2010). PAMPs are widely conserved in pathogens compared with the components of ETI. Thus, the PTI-mediated resistance is generally predicted to confer durable resistance (Liu et al., 2014). The rice *pi21* gene confers a non-race-specific resistance to the rice blast (Fukuoka and Okuno, 2001; Fukuoka et al., 2009). In this study, two *M. oryzae* isolates were used to investigate the common defense response pathways underlying *pi21*. The overlapped genes of Nip/GUY-11 and Nip/TMC-1 interactions may, at a certain extent, contain the genes involved in PTI or basal resistance. Thus, the overlapping genes that exhibited differential expression between two genotypes infected with both isolates may contribute to the loss-of-function of *Pi21*-mediated partial rice blast resistance.

### Preformed expression of defense-related genes in the *Pi21*-RNAi line

The expression levels of genes in the *Pi21*-RNAi line were compared with those in Nip at 0 hpi and 103 DEGs (86 up-regulated and 17 down-regulated) were identified to investigate the intrinsic differences between the resistant and susceptible genotypes. WEGO classification analysis showed that a high proportion of the DEGs belonged to the categories of “metabolic process,” “cellular process,” “catalytic activity,” and “response to stimulus.” Among the genes that were highly expressed in the *Pi21*-RNAi line, we detected significant enrichment in “plant—pathogen interaction,” “cutin, suberine, and wax biosynthesis,” and “flavone and flavonol biosynthesis” pathways. The enriched DEGs included those encoding receptor-like kinases, calcium-associated and calmodulin-related proteins, and a set of TFs. Previous studies showed that members of the LRR and SD subfamilies were implicated in pathogen defense response (Pastuglia et al., 2002; Albrecht et al., 2012). In this study, three RK genes, including LOC_Os09g19229.1 (LRR-Ia subfamily; RD class), LOC_Os11g40970.1 (LRR-XII subfamily; non-RD class), and LOC_Os04g53998.1 (SD-1c subfamily; RD class), were up-regulated in the *Pi21*-RNAi line compared with that in Nip at 0 hpi. Calcium influx is one of the earliest events upon pathogen recognition in plant defense response. Calcium-dependent protein kinases (CDPKs/CPKs) and calcium-related proteins play an important role in plant defense. The OsCPK10 (Fu et al., 2013) and OsCPK18 (Xie et al., 2014) were described as positive and negative regulators of *M. oryzae* resistance, respectively. Overexpression of *OsCPK4* gene enhances resistance to rice blast disease and the constitutive accumulation of OsCPK4 protein prepares rice plants for a rapid and potentiated defense response (Bundó and Coca, 2016). The CPK and cyclic-nucleotide gated channels genes (LOC_Os10g28240.1, LOC_Os02g50174.1, LOC_Os11g44680.1, and LOC_Os11g44680.1) were up-regulated as early as 0 hpi until later stages in the *Pi21*-RNAi line. This result indicates that Ca^2+^ participates in *Pi21* signaling transduction during early-stage defense response.

An auxin receptor *OsGH3.2* acted as a minor QTL in rice disease resistance. Activation of OsGH3.2 caused auxin-deficient morphological phenotypes and conferred broad-spectrum resistance against *M. oryzae* (Fu et al., 2011). *GH3.2* (LOC_Os01g55940.1) was up-regulated in *Pi21*-RNAi plants compared with that in Nip at 0 and 24 hpi. Wamaitha et al. (2012) have demonstrated that OsRap2.6 transcription factor belonging to ERF family contributes to rice innate immunity through its interaction with receptor for activated kinase-C1. In our study, *OsRap2.6* (LOC_Os04g32620.1) and other ERF gene LOC_Os09g11460.1 exhibited significantly higher level at 0 hpi and were more induced at 12 and 24 hpi in the *Pi21*-RNAi line compared with that in Nip. This result support previous study that *pi21*-mediated resistance might involve ethylene signaling (Fukuoka et al., 2015). A similar preformed expression feature of defense-related genes was also observed for partial resistance to rice blast by analyzing the expression of a set of 21 defense genes in 23 natural accessions displaying various levels of resistance to *M. oryzae* (Vergne et al., 2010). Our results suggest that a set of the defense responses induced in Nip on *M. oryzae* has been already active in the *Pi21*-RNAi line, which can allow plants to quickly respond to an aggressive and spreading pathogen.

Previous studies have also demonstrated that some NBS-LRR genes, such as the rice blast resistance genes *Pib* (Wang et al., 1999), *Pi5-1* (Lee et al., 2009), and *Pi-kh* (Sharma et al., 2005), are inducible upon pathogen infection. Li et al. (2015) performed comparative transcriptomic analysis of the durably resistant Digu and the susceptible Lijiangxintuanheigu and revealed a set of defense responses in Digu distinct from those mediated by NBS-LRR proteins. Bagnaresi et al. (2012) identified 75 resistance gene analogs as DEGs in the rice cultivar GV (compared with the susceptible rice line) and suggested that these resistance gene analogs were candidates contributing to the durable blast resistance in GV. We thus analyzed rice NBS-LRR DEGs in the *Pi21*-RNAi line and Nip. Six DEGs (LOC_Os08g19980.1, LOC_Os11g12000.1,LOC_Os11g11960.1,LOC_Os11g11580.1, LOC_Os12g17430.1, and LOC_Os08g20000.1) between the two genotypes at 0 hpi encode putative NBS-LRR proteins. Loc_Os11g11960.1 and Loc_Os12g17430.1 showed higher expression levels than that of others. However, Loc_Os12g17430.1 was suppressed on *M. oryzae* inoculation in both genotypes at later time points post-inoculation. Loc_Os11g11960.1 showed higher expression in the highly susceptible Nip than that in the *Pi21*-RNAi line. These NBS-LRR genes are unlikely to contribute to a resistance to both GUY-11 and TMC-1 strains. Second, 18 DEGs were identified based on keyword NBS-LRR or resistance protein out of the 1109 DEGs between the two rice genotypes. Among the 18 NBS-LRR DEGs, LOC_Os08g19980.1 and LOC_Os11g45180.1 were induced by *M. oryzae* infection and showed higher expression in the *Pi21*-RNAi line than that in Nip. These NBS-LRR genes are unlikely related to the *Pi21*-mediated blast resistance considering that the *Pi21*-RNAi line exhibits a slow and partial resistance to *M. oryzae* rather than an R gene-mediated hypersensitive response. However, rice NBS-LRR genes, such as *Pi35* (Fukuoka et al., 2014) and *Pi63* (Xu et al., 2014), confer durable blast resistance whose underlying mechanism may be associated with PTI. Further studies are needed to determine whether NBS-LRR genes function as an important partner of *Pi21* in the defense against *M. oryzae* infection.

### Defense-oriented reprogramming of genes in the *Pi21*-RNAi line during infection of *M. oryzae*

Plant immunity is governed by sophisticated systems involving perception receptors, signaling mediators, transcriptional regulators, and a line of anti-pathogen proteins (Jones and Dangl, 2006; Delteil et al., 2010; Liu et al., 2014). RKs are often responsible for perceiving internal and external signals. Most PRRs of PTI characterized to date are receptor-like kinases or receptor-like proteins (Kawano and Shimamoto, 2013). In this study, 43 RK genes were identified as DEGs between the *Pi21*-RNAi line and Nip. These RK genes encode proteins belonging to WAK, L-LEC, LRR, CAMK, MEKK, LRK10L-2, SD, DUF26, RLCK, and CR4L subfamilies. KEGG analysis indicated that most RK DEGs (35/43) were significantly enriched in “plant—pathogen interaction” pathway (Supplementary Table S9). Excluding PBS1 as a component of ETI, the other enriched RK DEGs were predicted to be involved in PTI. Thus, the RK DEGs identified might be associated with PTI underling *pi21* gene. WAK, LRR, and L-LEC play important roles as PRRs in PTI (Zipfel, 2014). Delteil et al. (2016) demonstrated that several wall-associated kinases participated positively and negatively in basal defense against rice blast fungus. The expression of *OsWAK112d* was repressed in both genotypes at 48 hpi. Moreover, our results showed that plasma membrane kinases *OsWAK59* was highly induced, whereas WAK/LRK10L-1 was less expressed in the *Pi21*-RNAi line compared with that in Nip as early as 12 hpi before the full maturation of *M. oryzae* appressorium. These wall-associated kinases may function as PRRs in basal defense against rice blast fungus.

*BAK1* is a ligand-independent coreceptor of receptor-like kinase RLKs, such as FLS2, EFR, and PEPR1 (Albrecht et al., 2012). *BAK1* also acts as a central regulator of PTI triggered by diverse PAMPs (Liu et al., 2014). In agreement with this condition, five BR signaling genes, *BAK1* (LOC_Os05g03450.1, LOC_Os12g03640.1, LOC_Os07g38800.1, LOC_Os07g38810.1, and LOC_Os05g17604.3), were all up-regulated in the *Pi21*-RNAi line compared with that in Nip at 24 hpi. Consistent with the expression pattern of *BAK1, FLS2* (LOC_Os12g12120.1) and *EFR* (LOC_Os11g40970.1, LP2) were also greatly induced in the *Pi21*-RNAi line at 24 hpi. The results suggested that high BAK1 activity together with *FLS2* and *EFR* in the *Pi21*-RNAi line may contribute to PTI mediated by the loss-of-function of *Pi21*.

Mitogen-activated protein kinase cascades represent candidates for downstream signaling processes during fungal infection, which are needed in both PTI and ETI (Kishi-Kaboshi et al., 2010; Bagnaresi et al., 2012). A MAPK cascade involving *OsMKK4* and the MAPKs *OsMPK3* and *OsMPK6* were shown to transduce chitin elicitor signal in rice defense responses (Kishi-Kaboshi et al., 2010). In our study, the induction of *OsMPK2, OsMPK4, OsMPK13*, and *OsMPK17* during *M. oryzae* infection was in partial agreement with these results reported by Reyna and Yang (2006). *M. oryzae*-induced expression of *OsMPK13* in this study was also observed by Wei et al. (2013). The *MPKKK1/OsEDR1* positively regulates rice resistance to *M. oryzae* (Shen et al., 2011). Consistent with these results, a significant induction of *OsMPKKK1* was observed in our data in both rice genotypes inoculated by two isolates. We observed that *MP3K55* (LOC_Os01g50400.1) belonging to non-RD was induced to higher level in the *Pi21*-RNAi line than that in Nip at 24 hpi challenged by both isolates. Non-RD kinase subclass is a molecular signature tightly associated with the immune response (Li et al., 2015). Therefore, we speculated that these differential regulated protein kinases may play crucial roles in the loss-of-function of *Pi21* resistance signaling.

Receptors and defense response genes are reported to contribute to QTL (Kou and Wang, 2010). In addition, PR genes are major defense-responsive genes. A number of reports have demonstrated that PR genes function at the late periods of plant defense response (Van Loon et al., 2006). In this study, multiple PR families were induced as early as 12 hpi in both genotypes. In addition, an increased accumulation of PR transcripts was observed at late stages (48 or 72 hpi). Out of the DEGs enriched in “metabolic process” and “response to stress” pathways, 62 PR genes were identified. Among these genes, three chitinase genes (LOC_Os01g18400.1, OsCHIT10; LOC_Os11g37960.1; and LOC_Os01g59840.1) and one β-1,3-glucanase gene (LOC_Os09g23084.1) were more highly expressed in the *Pi21*-RNAi line than that in Nip. Consistent with our findings, Bagnaresi et al. (2012) also observed the induction expression of β-1,3-glucanases and chitinase genes in the durable resistance genotype in response to *M. oryzae*. Chitin is the main component of the cell wall of *M. oryzae* and is known to be a kind of PAMP secreted by most pathogens. The highly induced β-1,3-glucanases and chitinase in the *Pi21*-RNAi line may block fungal development by degradation of β-1,3-glucans and chitin in fungi cell walls. Peroxidases contribute to fungal disease resistance by catalyzing lignin deposition and strengthening plant cell walls. Lipid transfer proteins (LTP) are involved in cutin synthesis, β-oxidation, and plant defense (Sels et al., 2008). In our study, 4 peroxidase genes (PR9) and 7 lipid-transfer protein (PR14) DEGs were more induced in the *Pi21*-RNAi line than Nip after *M. oryzae* inoculation. Phenylalanine ammonia lyase (PAL) is a key enzyme in the phenylpropanoid pathway. PAL is also involved in the synthesis of phytoalexins and lignin to prevent cell wall penetration by the pathogen. Tonnessen et al. (2015) reported that *OsPAL4* and possibly *OsPAL6* were key contributors to resistance governed by QTL and were potential breeding targets for improved broad-spectrum disease resistance in rice. The expression of *PAL6* was up-regulated in the *Pi21*-RNAi line relative to Nip as early as 24 hpi. The up-regulated PR genes may be associated with inhibiting fungal growth and spread infection through the degradation of the cell wall components (glucans, chitin, and proteins) of *M. oryzae*, as well as catalyzing lignin and cutin deposition of rice cell walls. These conditions are likely involved in the loss-of-function of *Pi21* partial resistance.

TFs play important roles in the regulation of defense-responsive genes (Cheng et al., 2015). The WRKY, AP2/ERF, bHLH, MADS, MYB, and NAC TF families have been reported to be involved in regulating rice defense responses to *M. oryzae* (Licausi et al., 2013; Yokotani et al., 2014). OsWRKYs are important regulators in rice blast resistance (Wei et al., 2013). WRKY13, WRKY22, WRKY30, WRKY45-1, WRKY45-2, WRKY47, WRKY53, and WRKY55 positively regulate rice defense response to *M. oryzae*, whereas WRKY28 and WRKY76 showed negative regulation (Cheng et al., 2015). The above mentioned WRKY genes were rapidly induced or repressed upon *M. oryzae* infection in this study. WRKY27, WRKY32, WRKY42, WRKY70, and WRKY93 were down-regulated in the *Pi21*-RNAi line after *M. oryzae* infection compared with those in Nip. In addition, KEGG enrichment analysis revealed that these five WRKY genes were involved in “plant—pathogen interaction” pathway. Cheng et al. (2015) reported that WRKY45-2, WRKY13, and WRKY42 form a transcriptional regulatory cascade and WRKY42 may negatively regulate rice resistance to *M. oryzae* by suppressing JA signaling-related genes. The expression of WRKY42 was much lower at five infection stages in the *Pi21*-RNAi line than that in Nip. This result suggests that low expression of WAKY42 may contribute to *pi21* partial resistance. NAC122, NAC131, and NAC111 were reported importantly in rice resistance to *M. oryzae* through regulation of the expression of defense-related genes and/or JA/ET signaling-related genes (Sun et al., 2013; Yokotani et al., 2014). The expression of five NAC TFs was faster and higher in the *Pi21*-RNAi line—*M. oryzae* interaction compared with that in the Nip—*M. oryzae* interaction during early stages of infection. This condition suggests potential roles of NAC TFs in *pi21* resistance. AP2/ERFs as another group of plant-specific transcription factors are involved in the signaling of ET/JA. AP2/ERFs also regulate the expression of some PR genes (Licausi et al., 2013). Two AP2/ERF genes *OsRap2.6* and LOC_Os09g11460.1 were more highly expressed in the *Pi21*-RNAi line relative to Nip. Taken together, multiple TF DEGs were identified in this study. WKRY, NAC, and AP2/ERF DEGs might be involved in the loss-of-function of *Pi21* rice blast resistance.

Besides RK, PR and TF genes, heavy metal—transport/detoxification protein domain genes were also identified in our data. *Pi21* has a putative heavy metal—transport/detoxification protein domain (Fukuoka et al., 2009), implying that metal transport by Pi21 might be associated with defense as previously reported (Stein et al., 2006; Liu et al., 2007). Some other reports indicate that a few Avr factors bind to the heavy metal associated domain of NBS-LRR proteins (Cesari et al., 2014). The data likely suggests an important role of this heavy metal binding domain in disease resistance. In this regard, we queried rice heavy metal—transport/detoxification protein domain (HMA; Pfam ID:PF00403) genes in the Rice Genome Annotation Project. There are 102 gene models (some genes containing more than one HMA domain) matched with Profile PF00403. Out of 1109 DEGs, five genes *Pi21* (LOC_Os04g32850.1), LOC_Os04g39300.1, LOC_Os01g20830.1, LOC_Os04g32030.1 and LOC_Os07g43040.1 with HMA domain(s) exhibited different expression pattern between the *Pi21*-RNAi line and Nip. The expression of *Pi21* was much lower at five infection stages in the *Pi21*-RNAi line than that in Nip. The expression of LOC_Os04g39300.1 was higher in the *Pi21*-RNAi line relative to Nip at 24 hpi while LOC_Os01g20830.1, LOC_Os04g32030.1 and LOC_Os07g43040.1 exhibited more expression at 72 hpi. Whether and how the heavy metal—transport/detoxification protein domain functions in disease resistance needs to be investigated further.

The plant hormones SA, JA, and ET function as signals in fine-tuning plant immune responses (Yang et al., 2013; De Vleesschauwer et al., 2016). We retrieved the genes related to SA, JA, and ET from the literature (Rzewuski and Sauter, 2008; Lyons et al., 2013) and the RiceCyc database (http://pathway.gramene.org/). Many of the genes were induced in the *M. oryzae*-infected rice plants. Among those genes, SA biosynthesis genes showed high transcript levels at 12 and 24 hpi, whereas JA- and ET-related genes were highly expressed at 12 and 72 hpi. Three SA biosynthesis genes (LOC_Os04g43800.1; LOC_Os02g41670.1; LOC_Os05g35290.1), six JA biosynthesis and response genes (*OsLOX1*, LOC_Os03g49380.1; *OsAOS2*, LOC_Os03g12500.1; *OsAOS3*, LOC_Os02g12680.1; *OsOPR1*, LOC_Os06g11290.1; *OsJAZ2*, LOC_Os07g05830.1; *OsJAZ11*, LOC_Os03g08320.1), and four ET-related genes were identified as DEGs between the two genotypes. SA in high plants can be synthesized via phenylalanine or isochorismate biosynthesis (Yang et al., 2013). KEGG analysis showed that LOC_Os04g15920.1, LOC_Os05g06970.1, LOC_Os04g58710.1, LOC_Os04g43800.1, and LOC_Os09g23540.1 were enriched in phenylpropanoid biosynthesis pathway at 24 hpi. Moreover, the expression levels of DEGs were higher in the *Pi21*-RNAi line than that in Nip. The results implicated that phenylpropanoid biosynthesis pathway may have an effect on partial blast resistance mediated by the loss-of-function of *Pi21*. The JA hormone is active regulators of rice immune responses (Liu et al., 2014). Lipoxygenases (LOX) are key enzymes in JA biosynthesis. JA ZIM-domain (JAZ) proteins in *Arabidopsis* and other plants act as transcriptional repressors of JA responses (Lyons et al., 2013). Our data showed that *LOX5* (LOC_Os03g49380.1) were up-regulated and *JAZ11* (LOC_Os03g08320.1) were down-regulated in the *Pi21*-RNAi line compared with that in Nip. The induction of JA signaling pathway might involved in the *pi21*-mediated resistance to *M. oryzae*. Ethylene was reported to be involved in rice basal immunity against the fungal infection (Iwai et al., 2006). Fukuoka et al. (2015) reported that application of an antagonist of ethylene biosynthesis before inoculation did not alter average lesion area in the plants containing *pi21*, whereas Aichiasahi plants carrying *Pi21* had significantly larger lesions than the corresponding controls, implying that *pi21*-mediated resistance involves ethylene signaling. In this study, four ET biosynthesis genes (*OsSAM2*, LOC_Os01g18860.1; *OsARD1*, LOC_Os10g28350.1; *ACS2*, LOC_Os04g48850.1; *ACS5*, LOC_Os01g09700.1) were identified as DEGs between the two genotypes. *OsSAM2* and *OsARD1* exhibited higher transcript levels continuously in the *Pi21*-RNAi line than that in Nip. *ACS5* and *OsACS2* were up-regulated and down-regulated in the *Pi21*-RNAi line compared with that in Nip at 72 hpi. However, how these DEGs function in the interaction between rice and *M. oryzae* needs to be investigated further. Taken together, the genes related to JA, ET, SA, and BR signaling pathways were induced or suppressed in the *M. oryzae*-inoculated *Pi21*-RNAi line. Moreover, these genes may play critical role in host resistance to *M. oryzae* infection.

## Conclusions

In this study, comparative analysis of the transcriptional profiles between the *Pi21*-RNAi transgenic rice line and Nip with *M. oryzae* infection uncovered distinctive regulation of defense responses against *M. oryzae* between the two genotypes. A total of 1109 genes were differentially expressed between the two genotypes with *M. oryzae* infection. GO and pathway analysis revealed that these DEGs were involved in metabolic process, cellular process, response to stimulus, catalytic activity, biosynthesis of secondary metabolites, plant—pathogen interaction, and plant hormone signaling transduction pathways. A set of DEGs were up-regulated in the *Pi21*-RNAi line compared with that in Nip at 0 hpi. Moreover, these genes were assigned to “plant—pathogen interaction,” “cutin, suberine, and wax biosynthesis,” and “flavone and flavonol biosynthesis” pathways. Of the genes assigned to plant—pathogen interaction, we identified 43 receptor kinase genes associated with pathogen-associated molecular pattern recognition and calcium ion influx. The expression levels of brassinolide-insensitive 1, flagellin sensitive 2 and elongation factor Tu receptor, and ET biosynthesis and signaling genes were higher in the *Pi21*-RNAi line than in Nip. This result suggested a robust PTI response in *Pi21*-RNAi plants and that JA/ET signaling was important in rice blast resistance. We also identified 53 transcription factor genes (including WRKY, NAC, DOF, and ERF families) and 62 PR genes that show differential expression between the two genotypes. This study highlights possible candidate genes that may play a role in the partial rice blast resistance mediated by the loss-of-function of *Pi21* and enhanced our understanding of the molecular mechanisms involved in the partial resistance against *M. oryzae*.

## Author contributions

This study was conceived by SQ. The experiments were performed by YZ, JZ, YL, ZY, HY, HH, CM, HQ. Data were analyzed by YZ. The paper was written by YZ and SQ. All authors have read and approved the final manuscript.

### Conflict of interest statement

The authors declare that the research was conducted in the absence of any commercial or financial relationships that could be construed as a potential conflict of interest.

## Supplementary material

Supplementary Table S1 **Primers used in this study**.

Supplementary Table S2 **Disease indexes of the ***Pi21***—RNAi line and Nipponbare inoculated with ***M. oryzae*** isolate TMC-1**.

Supplementary Table S3 **Summary of alignment statistics in 20 libraries referring to ***Oryza sativa*** L. ssp. ***japonica*** genome**.

Supplementary Table S4 **A total of 43,222 genes derived from 20 cDNA libraries**. The gene ID, RPKM of genes, description, KEGG, GO annotation, and Blast nr are presented in this table.

Supplementary Table S5 **Table listing 6865 differentially expressed genes (DEGs) between two genotypes infected with GUY-11 or TMC-1 infection**. We use FDR ≤ 0.001 and the absolute value of log2Ratio ≥ 1 as the threshold to judge the significance of gene expression difference. The gene ID, RPKM, GO and KEGG annotation of the genes are presented in the table.

Supplementary Table S6 **Table listing 1109 differentially expressed genes (DEGs) between two genotypes infected with GUY-11 and TMC-1 infection**. We use FDR ≤ 0.001 and the absolute value of log2Ratio ≥ 1 as the threshold to judge the significance of gene expression difference. The gene ID, RPKM, GO and KEGG annotation of the genes are presented in the table.

Supplementary Table S7 **GO enrichment analysis for 6865 DEGs. GO term classifications: C, cellular component; F, molecular function; P, biological process**. GO terms with *P* ≤ 0.05 were regarded as significantly enriched GO terms.

Supplementary Table S8 **KEGG enrichment analysis for 6865 DEGs between the ***Pi21***—RNAi line and Nipponbare**. KO pathways with ***Q***-value ≤ 0.05 were regarded as significantly enriched KO.

Supplementary Table S9 **List of 71 DEGs enriched in the “plant—pathogen interaction” KEGG pathway**. The gene ID, RPKM of genes, description, KEGG, GO annotation, and Blast nr are presented in this table.

Supplementary Table S10 **Detail of all 237 branch pathways in 35 MapMan major pathways**. The MapMan functional class, gene ID, RPKM of genes, and ratio of GUY-241-0 h vs. GUY-241-12 h are presented in the table.

Supplementary Table S11 **List of putative rice kinase genes in our results**. We queried rice kinase genes in the Rice Kinase Phylogenomics Database (http://ricephylogenomics.ucdavis.edu/kinase/index.shtml). All 63 rice kinase subfamilies were found in the result. A total of 1625 putative rice kinase genes are identified from the 43,222 expressed rice genes. Of the 1625 rice kinase genes, 43 genes showing significantly differential expression in the *Pi21*—RNAi line relative to Nip infected by two *M. oryzae* isolates are marked by ^*^. The IDs of the RK genes, the information on subfamily and class of the genes, RPKM, GO, and KEGG annotation of the genes are presented in the table.

Supplementary Table S12 **Expression pattern of 15 diterpene phytoalexin gene group**. The names of diterpene phytoalexin genes, gene ID, RPKM, GO, and KEGG annotation of the genes are presented in the table.

Supplementary Table S13 **List of putative transcription factor (TF) genes in our results**. We queried rice transcription factors genes in the Plant Transcription Factor Database (http://planttfdb.cbi.pku.edu.cn/index.php). All 55 transcription factor (TF) families were found in the result. A total of 1840 putative TF genes are identified in the 43,222 expressed rice genes. Of the 1840 putative TFs, 53 genes showing significantly differential expression in the *Pi21*—RNAi line relative to inoculated with two *M. oryzae* isolates are marked by ^*^. The TF family name, number, and RPKM are presented in the table.

Supplementary Table S14 **List of 47 DEGs enriched in the “plant hormone signaling transduction” KEGG pathway**. The gene ID, RPKM of genes, description, KEGG, GO annotation, and Blast nr are presented in this table.

Supplementary Table S15 **Pathogenesis-related genes in the rice genome. The names of PR genes are used according to Dou et al. (2014)**. Of the 1074 putative PR genes, 274 genes, which showed significantly differential expression in the *Pi21*—RNAi line relative to Nip, are marked by ^*^. The names of gene family, gene names, gene locus, gene ID, RPKM of genes, and gene annotation are presented in the table.

Supplementary Figure S1 **Expression level of ***Pi21*** between T_**1**_***Pi21***—RNAi plants and Nip**. Note: X-axis: Nip is the control plants. #104, 168, 178, 180, 192, 196, 202, 241, 251, 252, and 259 are transgenic *Pi21*—RNAi lines. Y-axis: relative expression of *Pi21* gene.

Supplementary Figure S2 **PCR confirmations for T-DNA flanking sequences in transgenic rice lines. (A)** Primers used for PCR amplification of T-DNA flanking sequences. LBR and RBF are primers specific for the flanking rice sequences surrounding different T-DNA integration site of each transgenic rice line. LSP2 and RB0b are primers specific for the T-DNA right or left border. **(B)** PCR product amplification with specific primers of T-DNA and rice genome sequences. Lanes 2–9: products amplified with primers LSP2/LBR. Lanes 10–17: products amplified with primers RB0b/RBF. Lane M: DNA molecular marker. For the #241 *Pi21*—RNAi line, the junctions between both T-DNA right and left borders and their flanking rice sequences were confirmed. This condition suggests that the #241 line carried a single T-DNA insertion.

Supplementary Figure S3 **RT-PCR validation of ***PR*** gene expression in #241 ***Pi21***—RNAi line and Nip infected with ***M. oryzae*** isolates**. This condition showed that the expression of the *PR* genes was induced from 12 hpi and indicated that the inoculation was successfully performed.

Supplementary Figure S4 **GO classification analysis of DEGs between the ***Pi21—***RNAi line and Nip at 12 (A)**, 24 **(B)**, 48 **(C)**, and 72 h **(D)**, respectively.

Supplementary Figure S5 **Validation of RNA-Seq data by qRT-PCR**. Fourteen DEGs involved in rice blast durable resistance were selected for validation and showed the same tendency with RNA-Seq. Data are represented as mean values ± S.D. for three replicates. *Asterisks* indicate a significant difference (^*^*P* < 0.05, ^**^*P* < 0.01 according to Paired *t*-test) between the *Pi21*-RNAi line and Nip at the same time point post-infected with the same isolate.
