# Supplementary material for: Transcriptome Analysis Highlights Defense and Signaling Pathways Mediated by Rice pi21 Gene with Partial Resistance to Magnaporthe oryzae
Source: Front Plant Sci. 2016 Dec 8;7:1834. doi: 10.3389/fpls.2016.01834 (PMC5143348; doi:10.3389/fpls.2016.01834)
Supplement: Supplementary Table S2 — Disease indexes of the Pi21—RNAi line and Nipponbare inoculated with M. oryzae isolate TMC-1. [file Table2.doc]

**Supplementary Table S2** Disease indexes of the *Pi21*-RNAi line and Nipponbare inoculated with *M. oryzae* isolate TMC-1

| plant | 1 | 2 | 3 | 4 | 5 | 6 | 7 | 8 | 9 | 10 | 11 | 12 | 13 | 14 | 15 | 16 | 17 | 18 | Mean |
| --- | --- | --- | --- | --- | --- | --- | --- | --- | --- | --- | --- | --- | --- | --- | --- | --- | --- | --- | --- |
| #241 | 4 | 2 | 4 | 4 | 3 | 2 | 4 | 4 | 5 | 4 | 4 | 5 | 5 | 4 | 4 | 4 | 4 | 5 | 3.94 |
| Nip | 5 | 5 | 4 | 6 | 6 | 4 | 5 | 6 | 7 | 7 | 6 | 6 | 6 | 6 | 6 | 5 | 5 | 6 | 5.61 |

Student's t-test: t= -5.81, p less than 0.0001

Note：rice seedlings were inoculated by spore-spray method（Li et al., 2015） and the disease index of each plant was evaluated according to the IRRI Standard Evaluation System of Rice (Chaudhary, R. C. 1996. Standard Evaluation System of Rice. (4th Edition), IRRI, Manila, Philippines, pp. 52)
