# Supplementary material for: Transcriptome Analysis Highlights Defense and Signaling Pathways Mediated by Rice pi21 Gene with Partial Resistance to Magnaporthe oryzae
Source: Front Plant Sci. 2016 Dec 8;7:1834. doi: 10.3389/fpls.2016.01834 (PMC5143348; doi:10.3389/fpls.2016.01834)
Supplement: Supplementary Table S3 — Summary of alignment statistics in 20 libraries referring to Oryza sativa L. ssp. japonica genome. [file Table3.doc]

Supplementary Table 3 Summary of alignment statistics in 20 libraries referring to *Oryza sativa* L. ssp*. japonica* genome

| Sample | Total Clean Reads | Total Mapped Reads | Unique Match | Multi-position Match | Total Unmapped Reads |
| --- | --- | --- | --- | --- | --- |
| GUY-*Pi21-RNAi*-0h | 10,642,019 | 9,384,795  (88.19%) | 8,760,657  (82.32%) | 624,138  (5.86%) | 1,257,224  (11.81%) |
| GUY-*Pi21-RNAi*-12h | 10,908,069 | 9,512,900  (87.21%) | 8,696,794  (79.73%) | 816,106  (7.48%) | 1,395,169  (12.79%) |
| GUY-*Pi21-RNAi*-24h | 10,666,277 | 9,472,462  (88.81%) | 8,785,762  (82.37%) | 686,700  (6.44%) | 1,193,815  (11.19%) |
| GUY-*Pi21-RNAi*-48h | 11,016,907 | 9,642,721  (87.53%) | 8,927,957  (81.04%) | 714,764  (6.49%) | 1,374,186  (12.47%) |
| GUY-*Pi21-RNAi*-72h | 11,133,573 | 9,682,616  (86.97%) | 8,885,918  (79.81%) | 796,698  (7.16%) | 1,450,957  (13.03%) |
| GUY-Nip-0h | 11,114,533 | 10,038,661  (90.32%) | 9,396,250  (84.54%) | 642,411  (5.78%) | 1,075,872  (9.68%) |
| GUY-Nip-12h | 10,605,837 | 9,436,902  (88.98%) | 8,750,916  (82.51%) | 685,986  (6.47%) | 1,168,935  (11.02%) |
| GUY-Nip-24h | 10,502,187 | 9,443,464  (89.92%) | 8,804,937  (83.84%) | 638,527  (6.08%) | 1,058,723  (10.08%) |
| GUY-Nip-48h | 11,369,315 | 10,214,891  (89.85%) | 9,453,310  (83.15%) | 761,581  (6.70%) | 1,154,424  (10.15%) |
| GUY-Nip-72h | 11,287,602 | 7,736,859  (68.54%) | 7,134,628  (63.21%) | 602,231  (5.34%) | 3,550,743  (31.46%) |
| TMC-*Pi21-RNAi*-0h | 11,179,207 | 9,739,262  (87.12%) | 9,046,437  (80.92%) | 692,825  (6.20%) | 1,439,945  (12.88%) |
| TMC-*Pi21-RNAi*-12h | 10,335,483 | 9,009,201  (87.17%) | 8,411,271  (81.38%) | 597,930  (5.79%) | 1,326,282  (12.83%) |
| TMC-*Pi21-RNAi*-24h | 10,908,281 | 9,561,360  (87.65%) | 8,866,363  (81.28%) | 694,997  (6.37%) | 1,346,921  (12.35%) |
| TMC-*Pi21-RNAi*-48h | 10,593,731 | 9,213,162  (86.97%) | 8,522,295  (80.45%) | 690,867  (6.52%) | 1,380,569  (13.03%) |
| TMC-*Pi21-RNAi*-72h | 10,454,329 | 7,860,319  (75.19%) | 7,298,401  (69.81%) | 561,918  (5.37%) | 2,594,010  (24.81%) |
| TMC-Nip-0h | 11,085,204 | 9,964,172  (89.89%) | 9,296,308  (83.86%) | 667,864  (6.02%) | 1,121,032  (10.11%) |
| TMC-Nip-12h | 10,932,514 | 9,789,685  (89.55%) | 9,111,741  (83.35%) | 677,944  (6.20%) | 1,142,829  (10.45%) |
| TMC-Nip-24h | 10,466,548 | 9,256,671  (88.44%) | 8,404,461  (80.30%) | 852,210  (8.14%) | 1,209,877  (11.56%) |
| TMC-Nip-48h | 11,115,658 | 9,869,299  (88.79%) | 9,145,909  (82.28%) | 723,390  (6.51%) | 1,246,359  (11.21%) |
| TMC-Nip-72h | 10,838,876 | 8,221,184  (75.85%) | 7,597,515  (70.10%) | 623,669  (5.75%) | 2,617,692  (24.15%) |
