# Supplementary figures and images for: Transcriptome Analysis Highlights Defense and Signaling Pathways Mediated by Rice pi21 Gene with Partial Resistance to Magnaporthe oryzae
Source: Front Plant Sci. 2016 Dec 8;7:1834. doi: 10.3389/fpls.2016.01834 (PMC5143348; doi:10.3389/fpls.2016.01834)

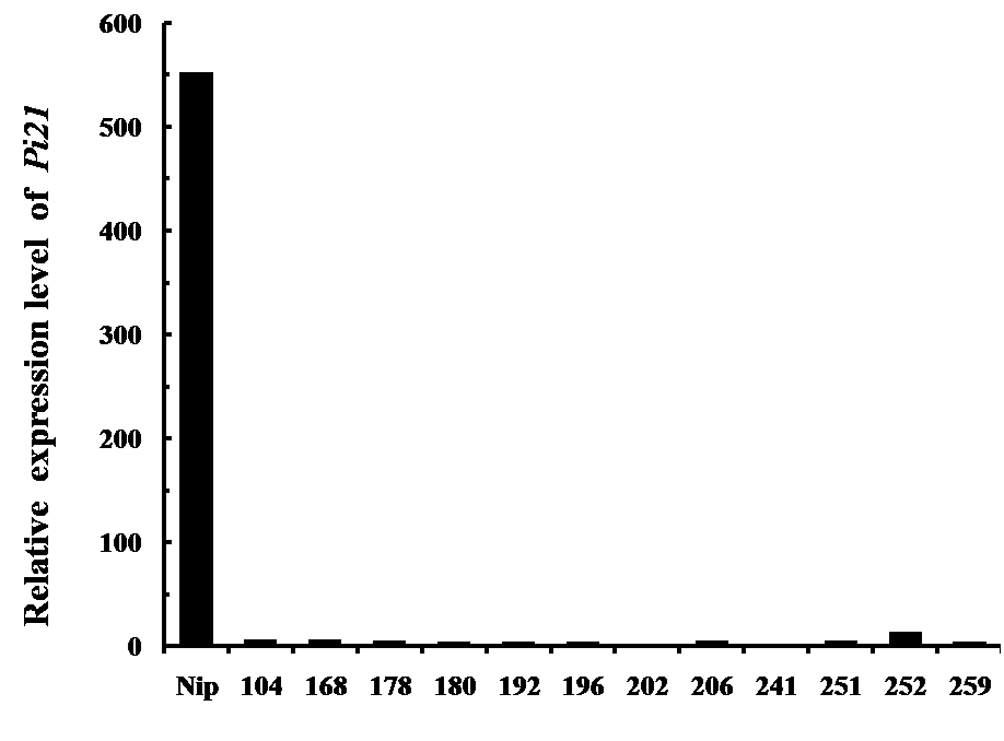

Supplement: Supplementary Figure S1 — Expression level of Pi21 between T1Pi21—RNAi plants and Nip. Note: X-axis: Nip is the control plants. #104, 168, 178, 180, 192, 196, 202, 241, 251, 252, and 259 are transgenic Pi21—RNAi lines. Y-axis: relative expression of Pi21 gene. [file Image1.tif]

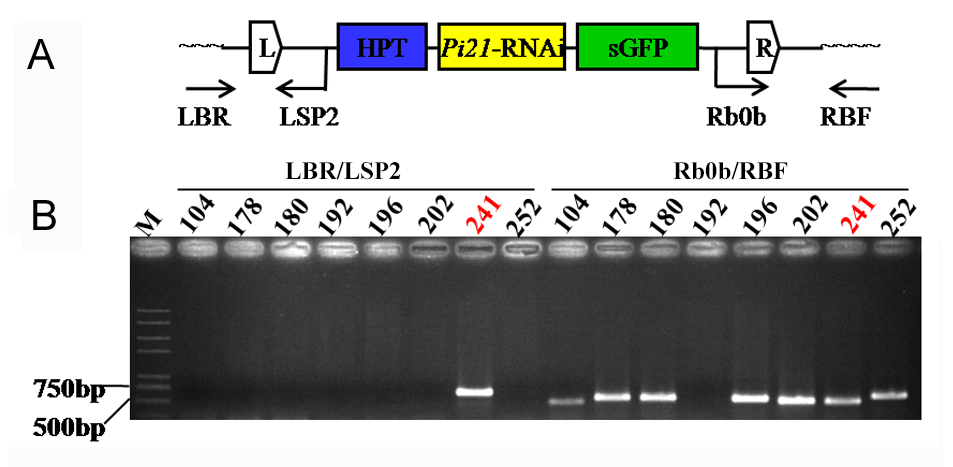

Supplement: Supplementary Figure S2 — PCR confirmations for T-DNA flanking sequences in transgenic rice lines. (A) Primers used for PCR amplification of T-DNA flanking sequences. LBR and RBF are primers specific for the flanking rice sequences surrounding different T-DNA integration site of each transgenic rice line. LSP2 and RB0b are primers specific for the T-DNA right or left border. (B) PCR product amplification with specific primers of T-DNA and rice genome sequences. Lanes 2–9: products amplified with primers LSP2/LBR. Lanes 10–17: products amplified with primers RB0b/RBF. Lane M: DNA molecular marker. For the #241 Pi21—RNAi line, the junctions between both T-DNA right and left borders and their flanking rice sequences were confirmed. This condition suggests that the #241 line carried a single T-DNA insertion. [file Image2.tif]

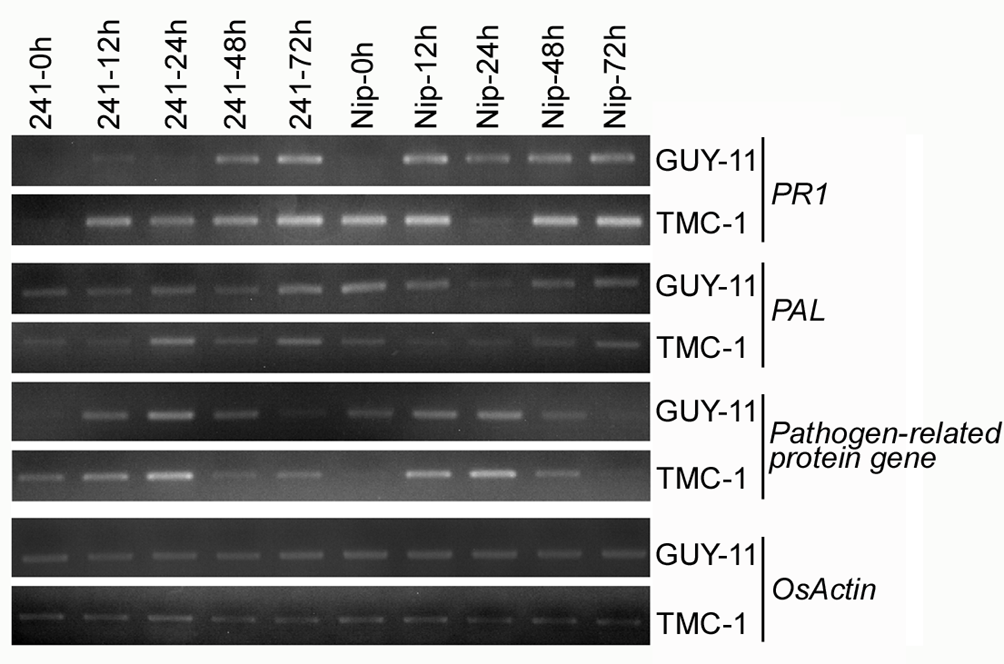

Supplement: Supplementary Figure S3 — RT-PCR validation of PR gene expression in #241 Pi21—RNAi line and Nip infected with M. oryzae isolates. This condition showed that the expression of the PR genes was induced from 12 hpi and indicated that the inoculation was successfully performed. [file Image3.tif]

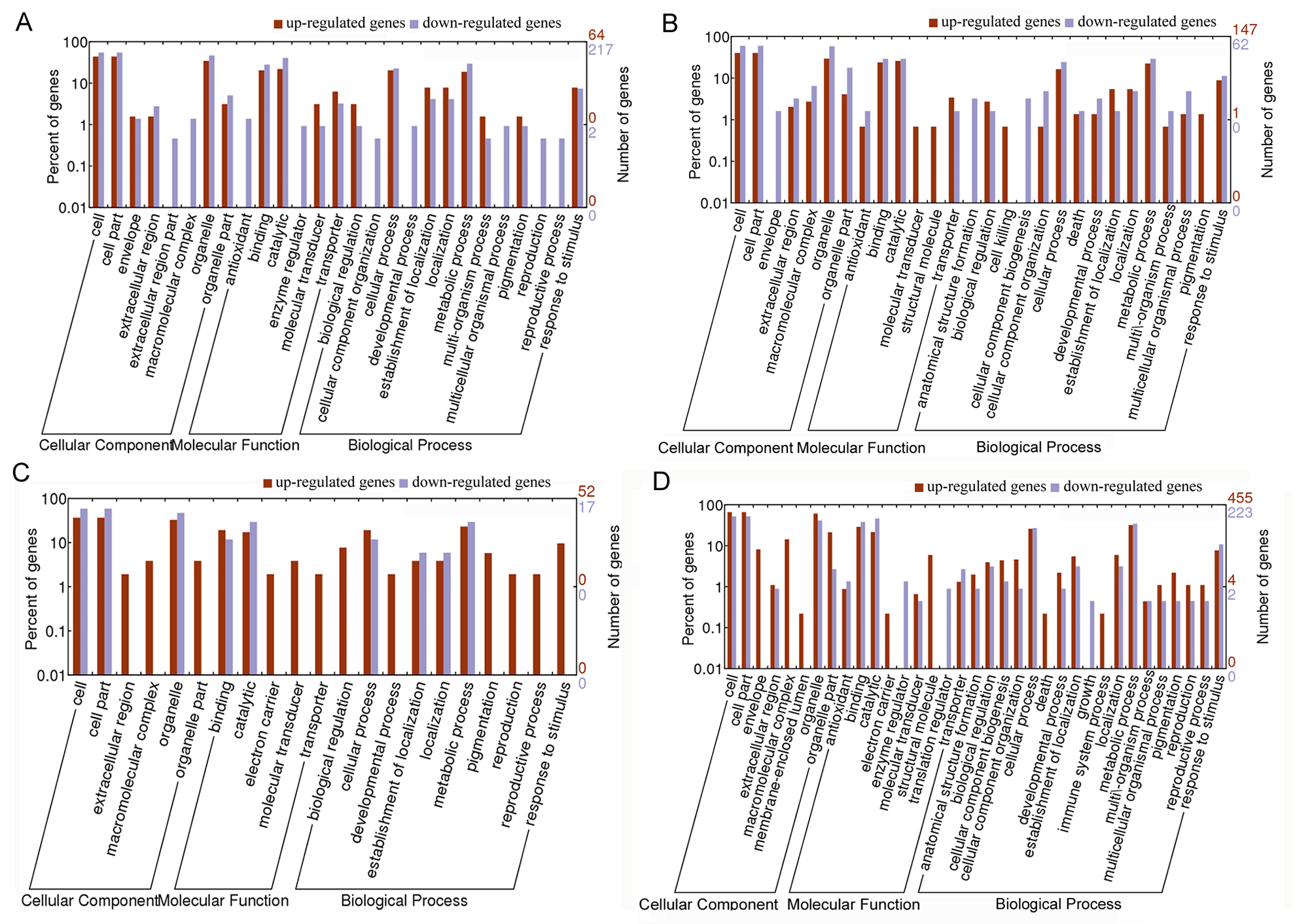

Supplement: Supplementary Figure S4 — GO classification analysis of DEGs between the Pi21—RNAi line and Nip at 12 (A), 24 (B), 48 (C), and 72 h (D), respectively. [file Image4.tif]

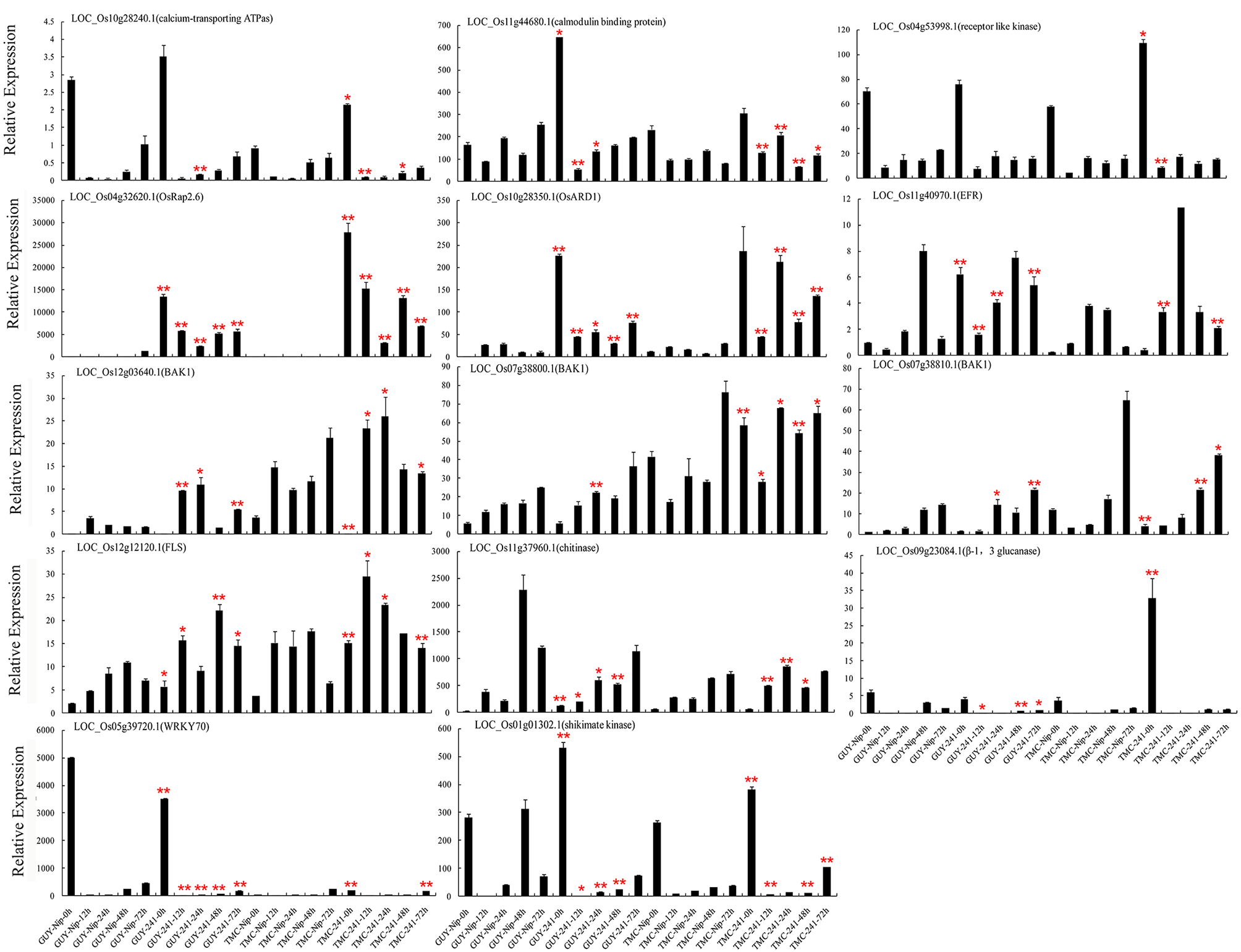

Supplement: Supplementary Figure S5 — Validation of RNA-Seq data by qRT-PCR. Fourteen DEGs involved in rice blast durable resistance were selected for validation and showed the same tendency with RNA-Seq. Data are represented as mean values ± S.D. for three replicates. Asterisks indicate a significant difference (*P < 0.05, **P < 0.01 according to Paired t-test) between the Pi21-RNAi line and Nip at the same time point post-infected with the same isolate. [file Image5.tif]
